# Cognitive deficits in schizophrenia: an updated metanalysis of the scientific evidence

**DOI:** 10.1186/1471-244X-12-64

**Published:** 2012-06-20

**Authors:** Mario Fioravanti, Valentina Bianchi, Maria Elena Cinti

**Affiliations:** 1 Department of Neurology and Psychiatry, University of Rome Sapienza, P.le A. Moro 5, Rome, 00185, Italy

**Keywords:** Schizophrenia, Cognitive deficits, Memory, Attention, Executive function, Language, IQ, Meta-analysis

## Abstract

**Background:**

This is an update of a previous meta-analysis published in 2005.

**Methods:**

It includes the data published up to march 2010 for a total of 247 papers and 18,300 cases. Cognitive deficits are examined in 5 different domains: Memory functioning (128 studies), Global cognitive functioning (131 studies), Language (70 studies), Executive function (67 studies), Attention (76 studies). Only controlled studies were included: patients vs. normal subjects.

**Results:**

Results evidence that in all domains and in all different analyses performed within each domain, patients show a significant reduction of cognitive efficiency with respect to normal subjects. The between studies heterogeneity is very high in almost all domains. There are various sources of this heterogeneity (age, sex, sample size, type of patients, and type of measurement) which contribute to the high degree of not-overlapping information offered by the single studies.

**Conclusions:**

Our results, based on the current scientific evidence, confirm the previous findings that there is a generalized impairment of various cognitive functions in patients with schizophrenia when compared to normal cases. The modalities with which these results are obtained have not changed over the years and the more recent studies do not modify the high heterogeneity previously found between the studies. This reduces the methodological quality of the results. In order to improve the methodological quality of the studies performed in the field of cognitive deficits of patients with schizophrenia, various factors should be taken into account and better managed in designing future studies.

## Background

There is a vast scientific evidence, accumulated in several years of research, that the cognitive functioning of patients with schizophrenia is characterized by deficits [1]. An early hypothesis was that these cognitive deficits might have a progression over time and depend on the length of disease [2].

More recent evidence indicates that the severity of cognitive deficits of patients with schizophrenia is related to age of onset (deficits of patients with early onset are more severe than those of patients with a late onset) while, the subsequent length of disease does not add further deterioration to the deficits already present at the early stages [3,4].

Many other studies have made a link between the functional disability of these patients and their cognitive impairment [5] but, at the same time, they have put in evidence the heterogeneous distribution of cognitive deterioration in this population of patients [6].

As a whole, the scientific production concerning the cognitive problems linked to schizophrenia is very large and prolonged across many decades. Our search, performed 5 years ago on this topic, found 1,275 papers published up to that time on schizophrenia and cognitive deficits [7] and even more papers were published in the following 5 years.

There are many problematic hypotheses and uncertainties about the meaning and the origins of the cognitive problems associated with schizophrenia, and these are still waiting for an answer despite the high and still growing trend of the scientific production in this area. It is unclear if there are specific cognitive problems due to schizophrenia or if they may be linked to intervening and concomitant factors during the chronic development of the disease. These factors could be age, differences between clinical forms, concomitant treatments, or severity and length of disease [4].

A common conclusion offered by the different studies concerning the various aspects of schizophrenia and cognitive deficits, is the presence of high heterogeneity of results. Partially, this heterogeneity is due to methodological problems such as the relative small number of cases of most of the studies, the often unclear characterization of patients and their clinical history, and the systematic unbalance between the number of patients and that of the control groups [7].

Another component of heterogeneity is due to the clinical diversity of patients included in the different studies where groups are composed of inpatients in some instances and of outpatients in other instances, while the majority of studies present results obtained from an unspecified mixture of both types of patients. In some other studies groups of patients with different length of disease or with different types of therapies are indiscriminately put together.

The third component of heterogeneity is the statistical heterogeneity which includes the chance component of variance plus the other components due to possible specific sources. This part of heterogeneity should be the one which with appropriate procedures could be explored, but this possibility will be real only after the offsetting of the obscuring interference of the methodological and clinical heterogeneity.

The previous meta-analysis that we performed in 2005 was based on distinct cognitive areas of which deficits were analyzed in separate tables: memory deficits, IQ deterioration, language deficits, executive functioning deficits, and attention deficits. The presence of a cognitive impairment was found in all cognitive areas, but it was not possible to overcome the prevalent component of the methodological and clinical heterogeneity which emerged from the results.

The present work is an update, after five years, of the previous meta-analysis and incorporates all the data produced after the previous review up to March 2010. The more recent data-sources were identified with the same criteria used in the previous systematic review.

This meta-analysis principally aims to evaluate the presence of cognitive differences between patients with schizophrenia and normal cases. No specific cognitive area is addressed or excluded and those described in this meta-analysis are empirically defined according to the prevalent themes of the current scientific literature. The secondary aim in updating the previous work was to control for the stability of results in comparison to the results obtained in the previous meta-analysis and the methodological quality of the studies.

## Methods

In order to perform the update of the previous review the following databases were searched, PubMed, PsycInfo, PsycArticles by these keywords: ‘cognitive deficit*’ AND ‘schizophrenia patients’; ‘controlled study’. Only data obtained by human subjects and identifiable until March 2010 were included. No restrictive language selection criteria were applied.

A total of 1,219 works were identified including 700 papers from PubMed, and 654 from PsychInfo and PsycArticles. The 135 papers found from multiple sources were considered only once.

All papers examined for inclusion concerned controlled studies with human subjects where patients with schizophrenia were compared to normal subjects in terms of cognitive functioning. The decision for inclusion was taken by consensus between two of the authors. In case of disagreement, a third author was called in to give his judgment.

**Figure 1:**
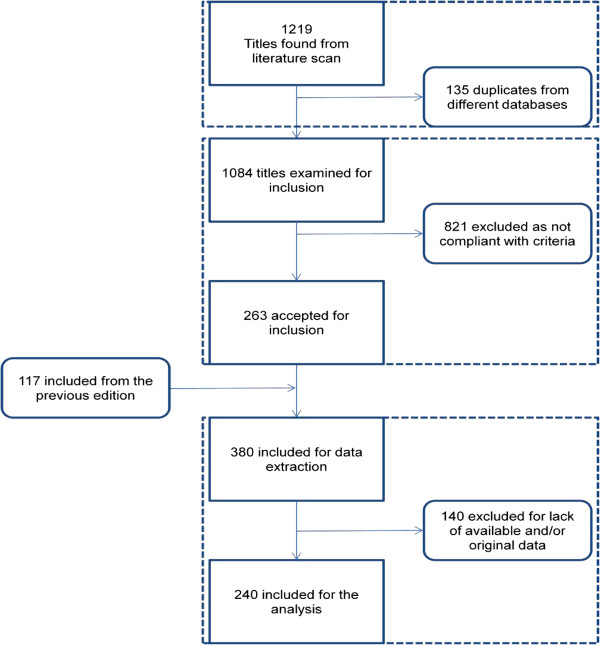
Flow chart of studies.

After this search, 270 papers were eligible to be added to the 117 already included to the previous systematic review (see Figure 1).

Some of the excluded studies were found lacking of results expressed in numerical form and their data could not be retrieved even when the authors were contacted for this reason (a curious example of this lack of numerical data is constituted by several papers published by the same Journal of which the editorial guidelines required by the authors to avoid to put numerical tables in their papers); other papers were excluded because they did not conform to the inclusion criteria for this meta-analysis. In particular, we excluded all the studies where the inclusion of patients was done by selecting the cases on the basis of a specific stratification by IQ level.

In the following phase, consisting of the data extraction, more papers were excluded from the 270 already found compliant with the inclusion criteria, when they were found part of a series of partial data publications all concerned with the same study. In these cases, in order to avoid an unsuitable redundancy of the data included, we accepted only the data which were found in the most recent of the publications or which indicated the largest number of cases among the other papers of the same series.

In conclusion of the data extraction process, 123 new papers were added to the 117 ones already present in the previous systematic review for a total of 240 papers. Each study may have offered data to one (111 papers) or more than one (129 papers) domains examined in the review.

The analysis of data is articulated on different cognitive domains. This organization is maintained the same as in the previous meta-analysis, since it still represents the most inclusive way of ordering the different types of measurement prevalently used in this field. As a confirmation of this, we have found a still growing number of papers which were carried out in the last years with modalities of cognitive assessment compatible with this organization of cognitive domains.

The distribution of the included papers in the various tables concerning the areas of cognitive functioning is:

1. Memory functioning (128 studies),

2. Global cognitive functioning (131 studies),

3. Language (70 studies),

4. Executive function (67 studies),

5. Attention (76 studies).

The different areas of cognitive functioning are articulated in different analyses as follows (see Table 1):

Memory Functioning

**Figure 2:**
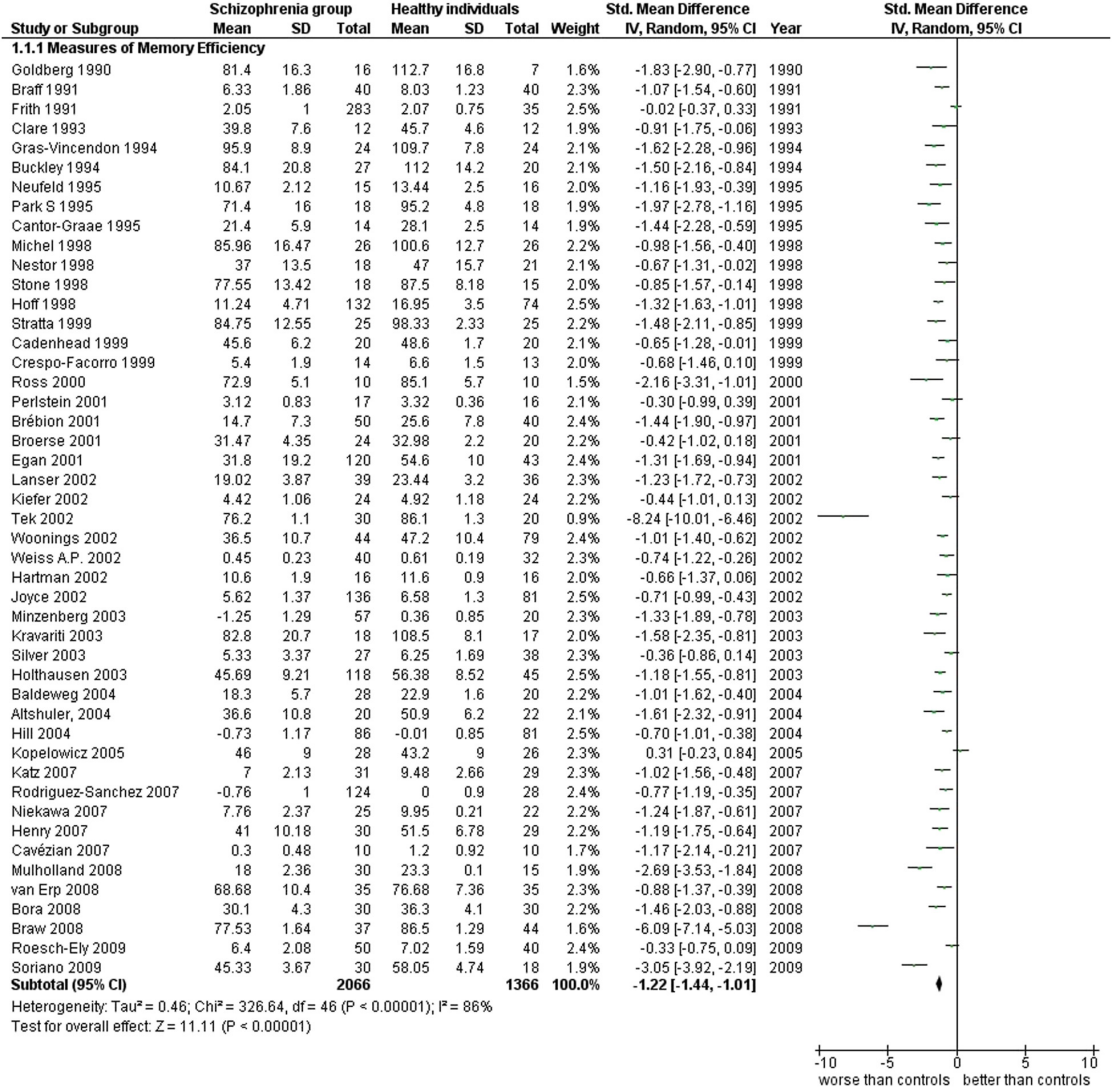
Measures of Memory Efficiency.

**Figure 3:**
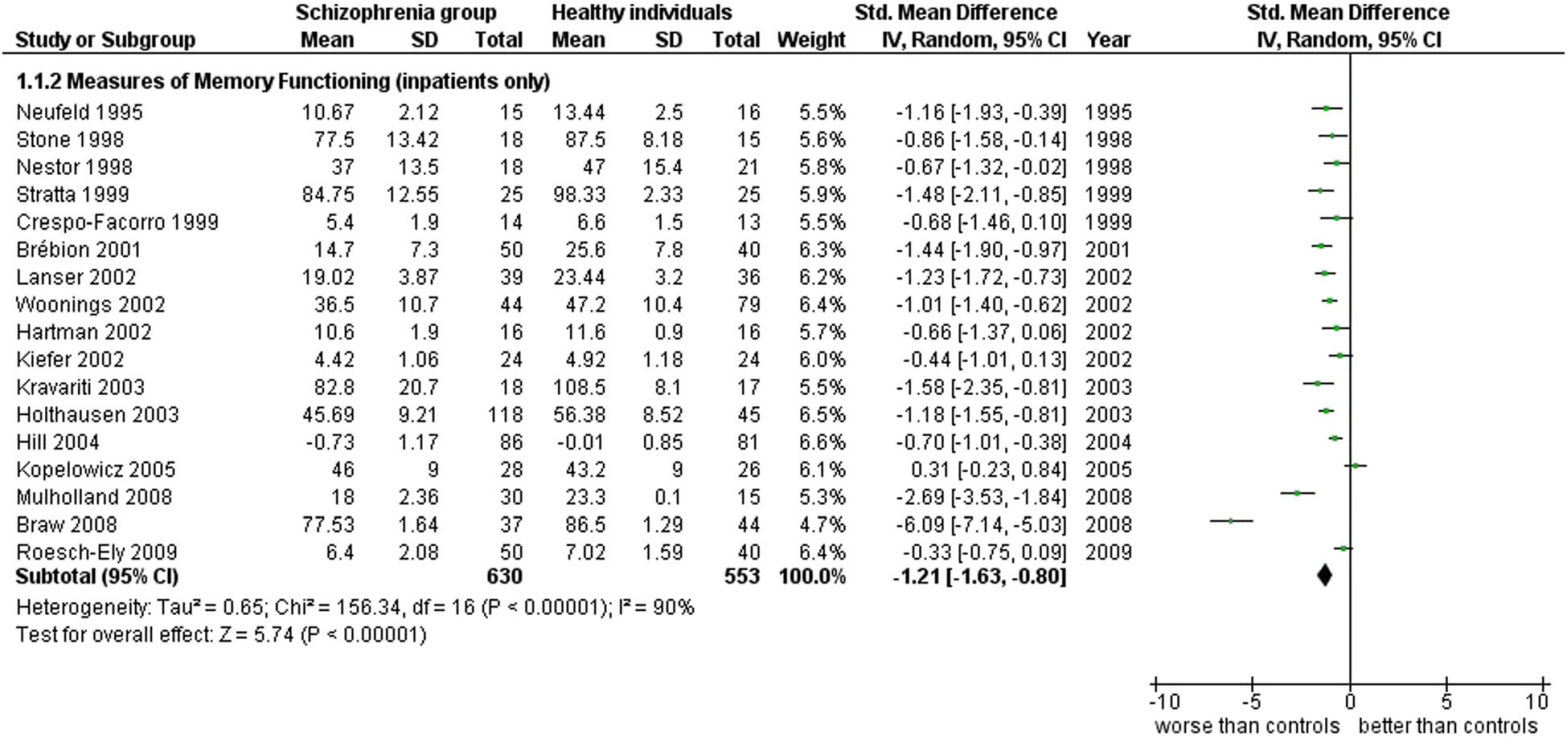
Measures of memory functioning (inpatients only).

**Figure 4:**
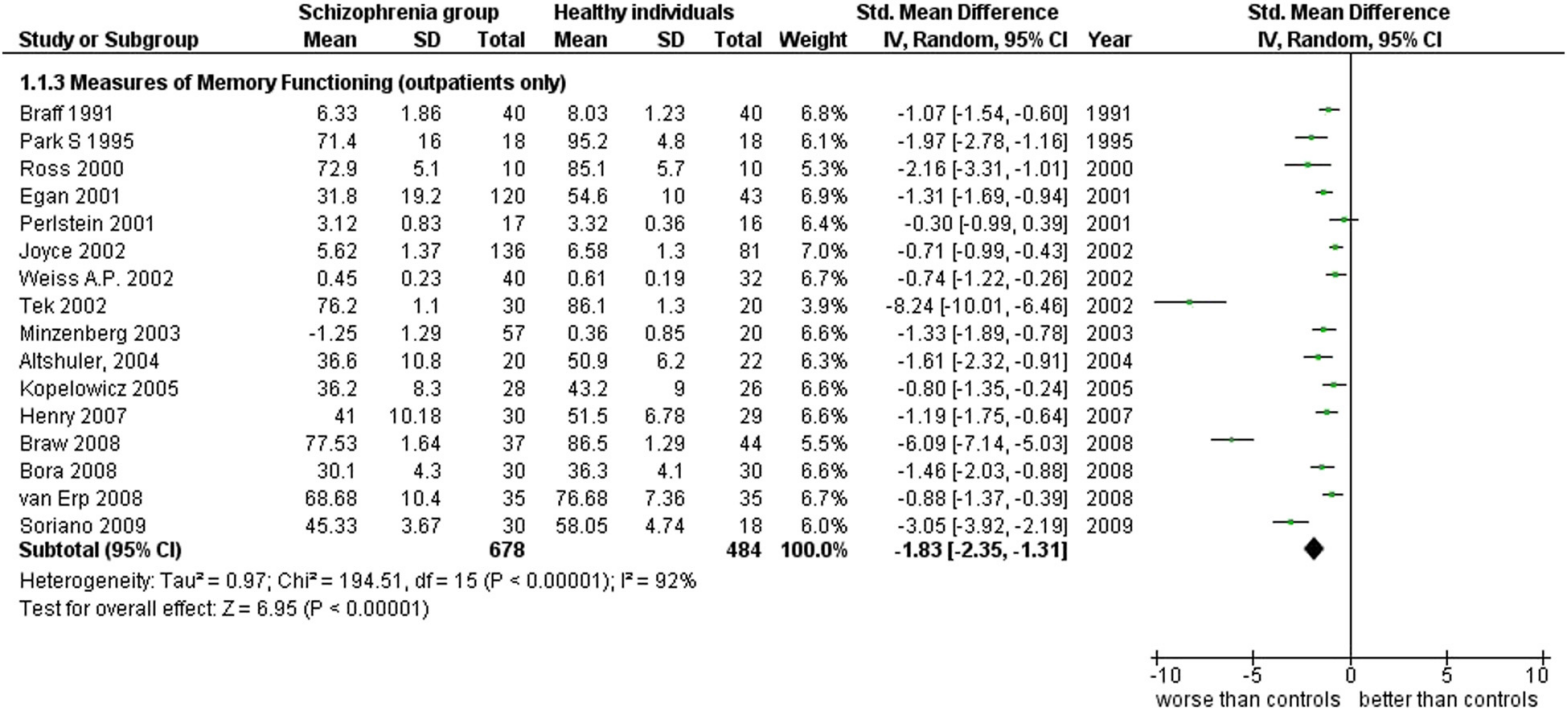
Measures of memory functioning (outpatients only).

**Figure 5:**
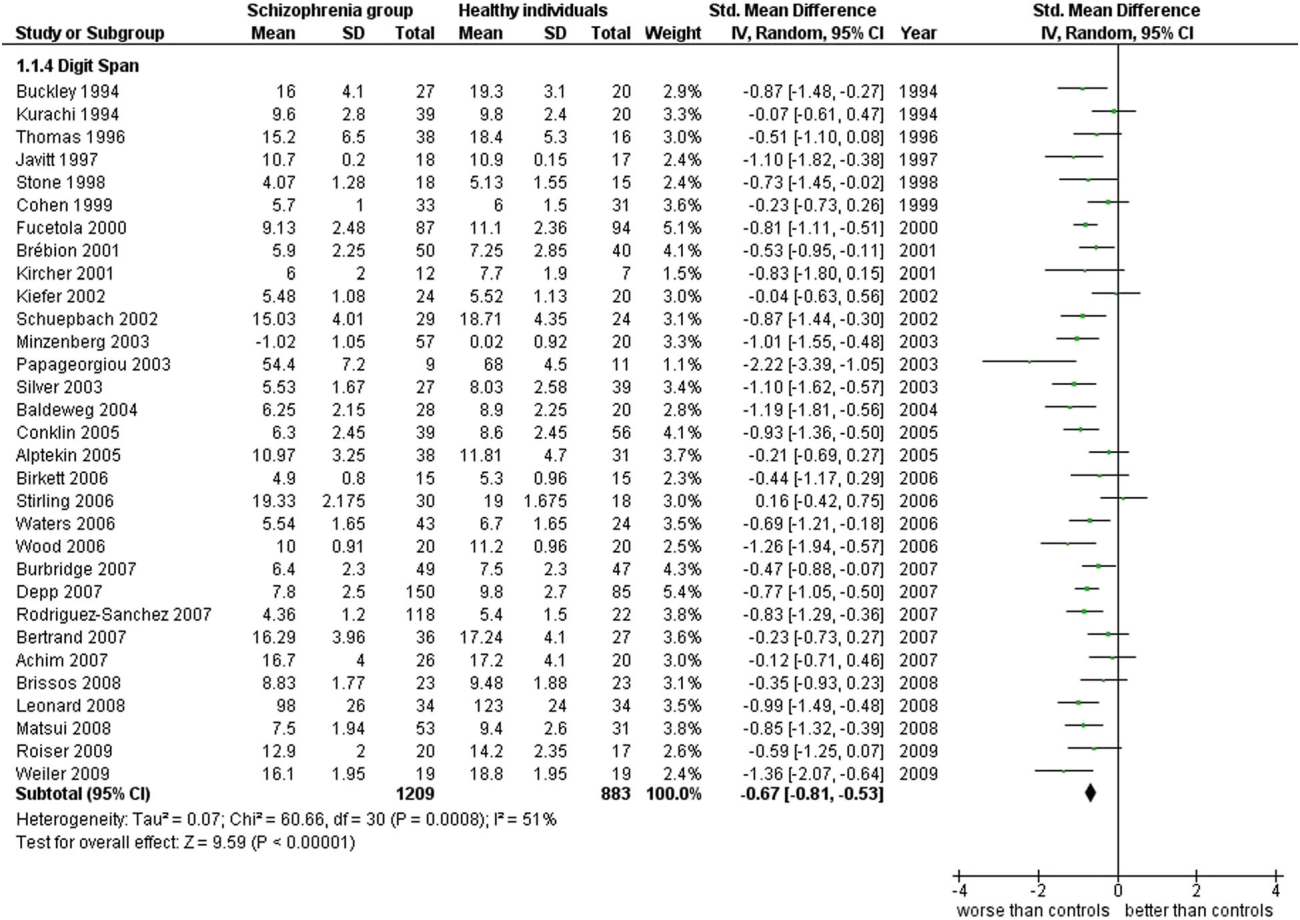
Digit span.

**Figure 6:**
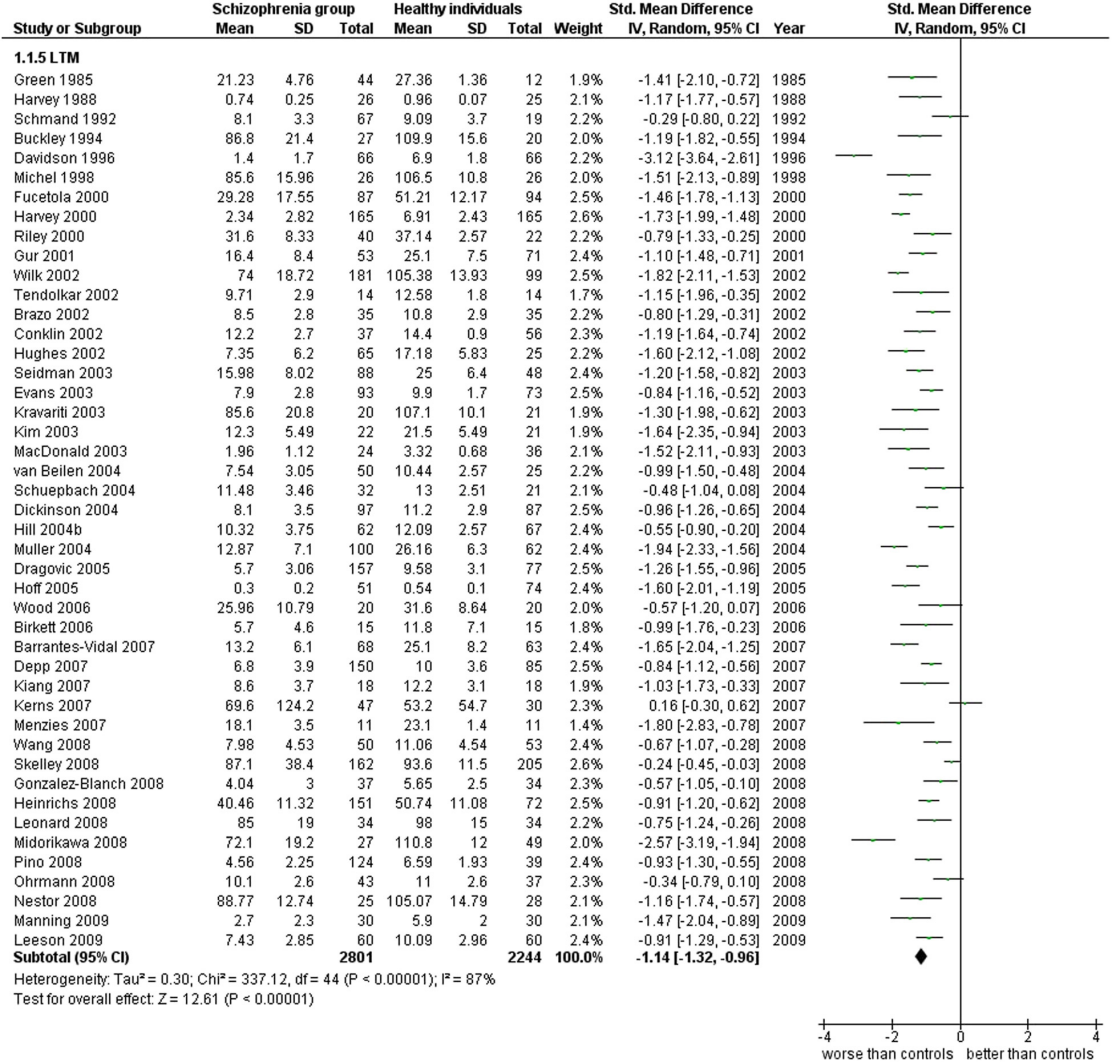
Long term memory.

**Figure 7:**
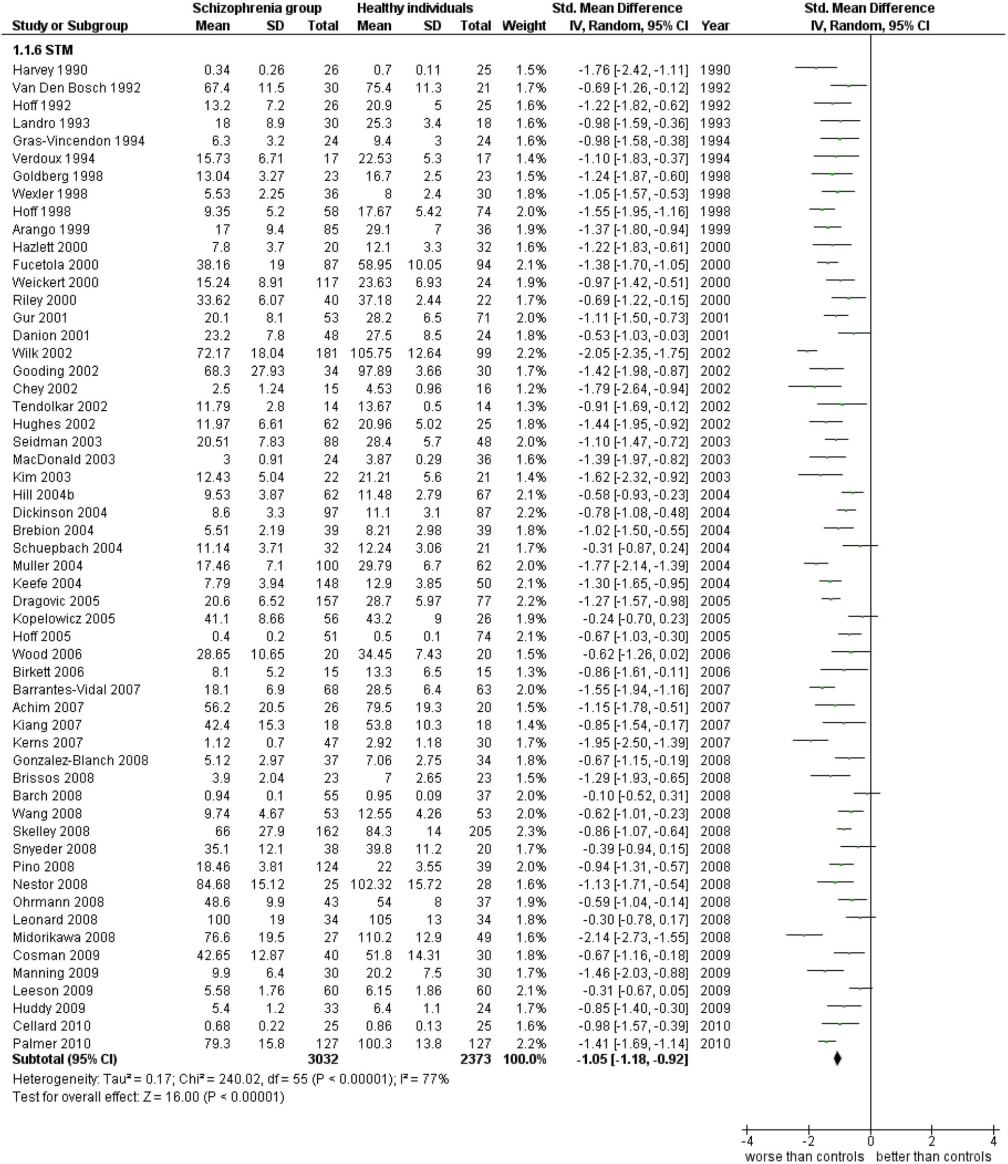
Short term memory.

Measures of Memory Efficiency (47 studies). This analysis includes all data from papers where there wasn’t a more specific distinction between types of memory characteristic to be examined (see Additional file 1: Table S1 and Figure 2).

Measures of Memory Functioning (inpatients only) (17 studies). This analysis includes only those studies from Additional file 1: Table S1 which were specified to be performed with inpatients (see Additional file 1: Table S1 and Figure 3).

Measures of Memory Functioning (outpatients only) (16 studies). This analysis includes only those studies from the Additional file 1: Table S1 which were specified to be performed with outpatients (see Additional file 1: Table S1 and Figure 4).

Digit Span (31 studies). This analysis includes only those studies which were performed by the Digit Span as a measure of immediate memory (see Additional file 1: Table S1 and Figure 5).

LTM (45 studies). This analysis includes only those studies which were specified to be performed with measures of long term memory (see Additional file 1: Table S1 and Figure 6).

STM (56 studies). This analysis includes only those studies which were specified to be performed with measures of short term memory (see Additional file 1: Table S1 and Figure 7).

Global cognitive functioning

**Figure 8:**
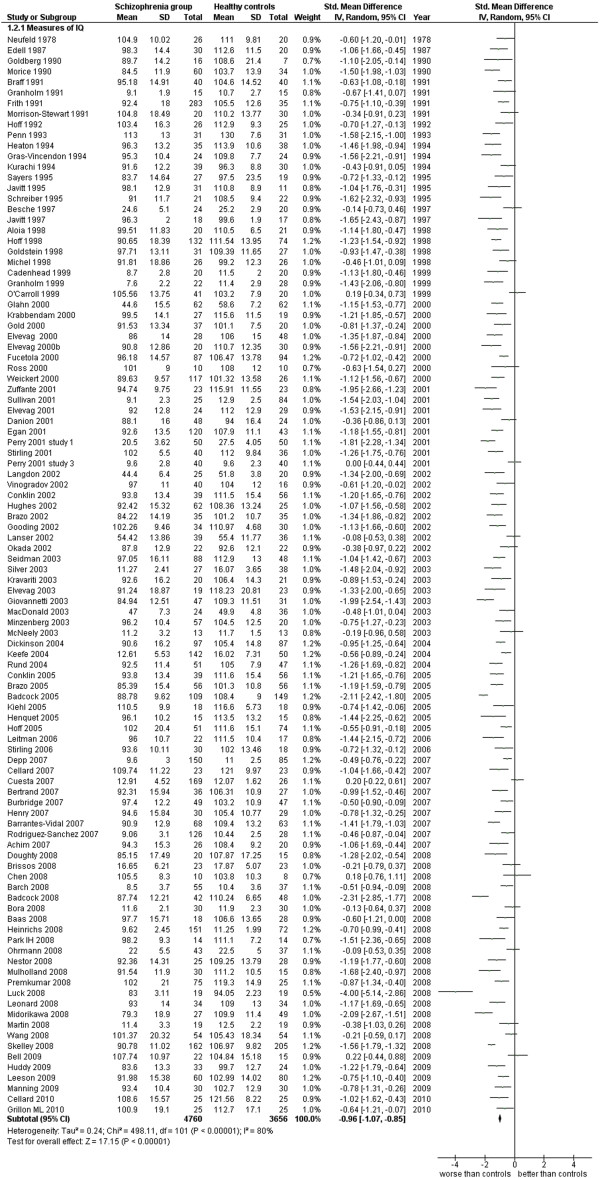
Measures of IQ.

**Figure 9:**
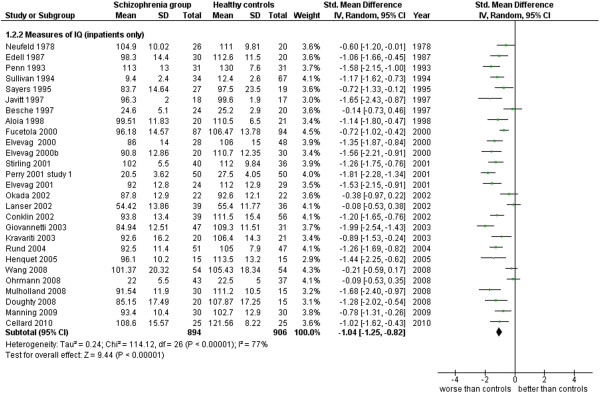
Measures of IQ.

**Figure 10:**
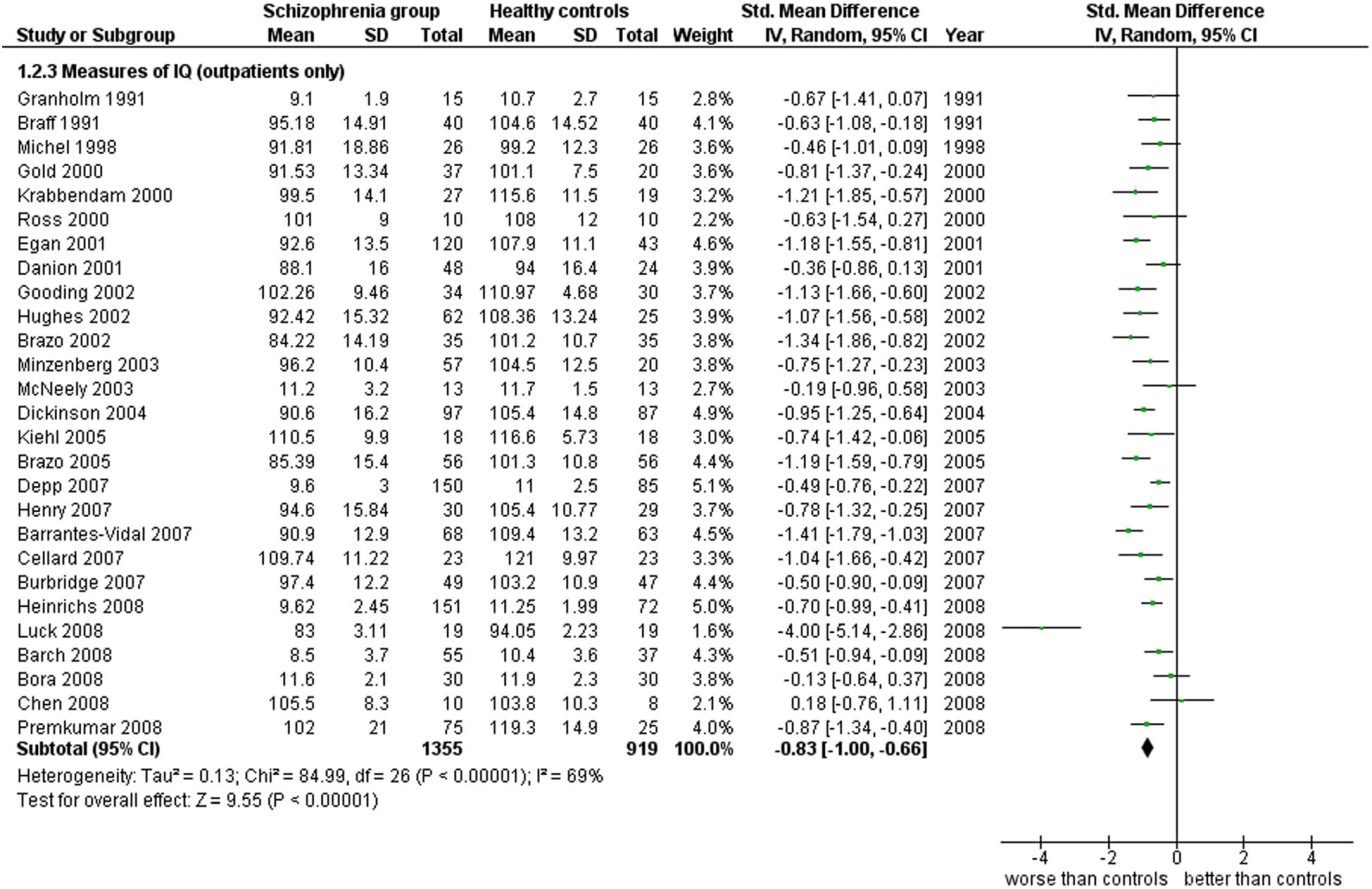
Measures of IQ (outpatients only).

**Figure 11:**
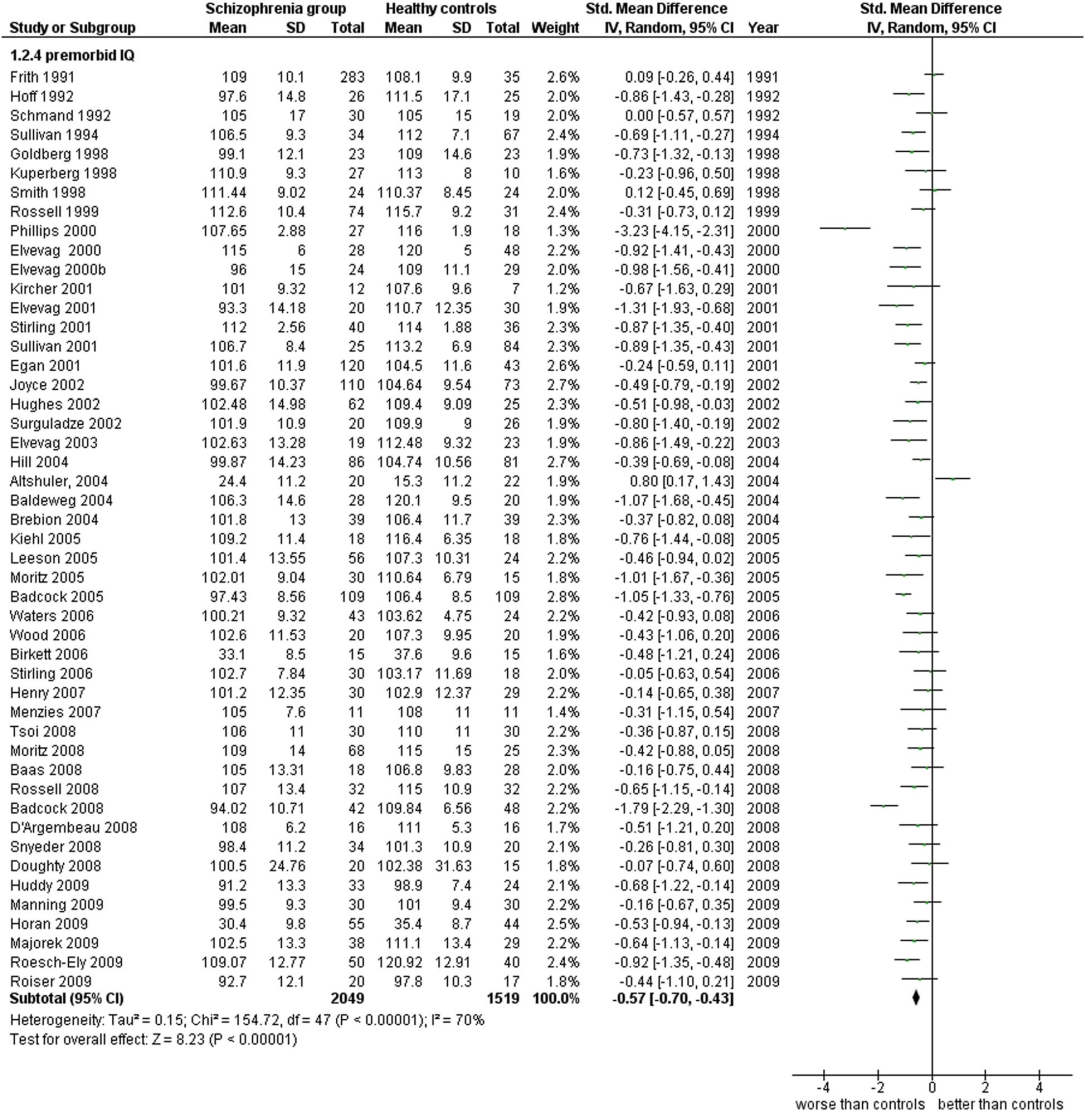
Premorbid IQ.

Measures of IQ (102 studies). This analysis includes only those studies which were specified to be performed with measures of IQ or with measures of general intelligence (see Additional file 1: Table S1 and Figure 8).

Measures of IQ (inpatients only) (27 studies). This analysis includes only those studies from Additional file 1: Table S1.1 which were specified to be performed with inpatients (see Additional file 1: Table S1 and Figure 9).

Measures of IQ (outpatients only) (27 studies). This analysis includes only those studies from Additional file 1: Table S1.1 which were specified to be performed with outpatients (see Additional file 1: Table S1 and Figure 10).

Premorbid IQ (48 studies). This analysis includes only those studies which were specified to be performed with measures of premorbid IQ as described by the authors (see Additional file 1: Table S1 and Figure 11).

Language

**Figure 12:**
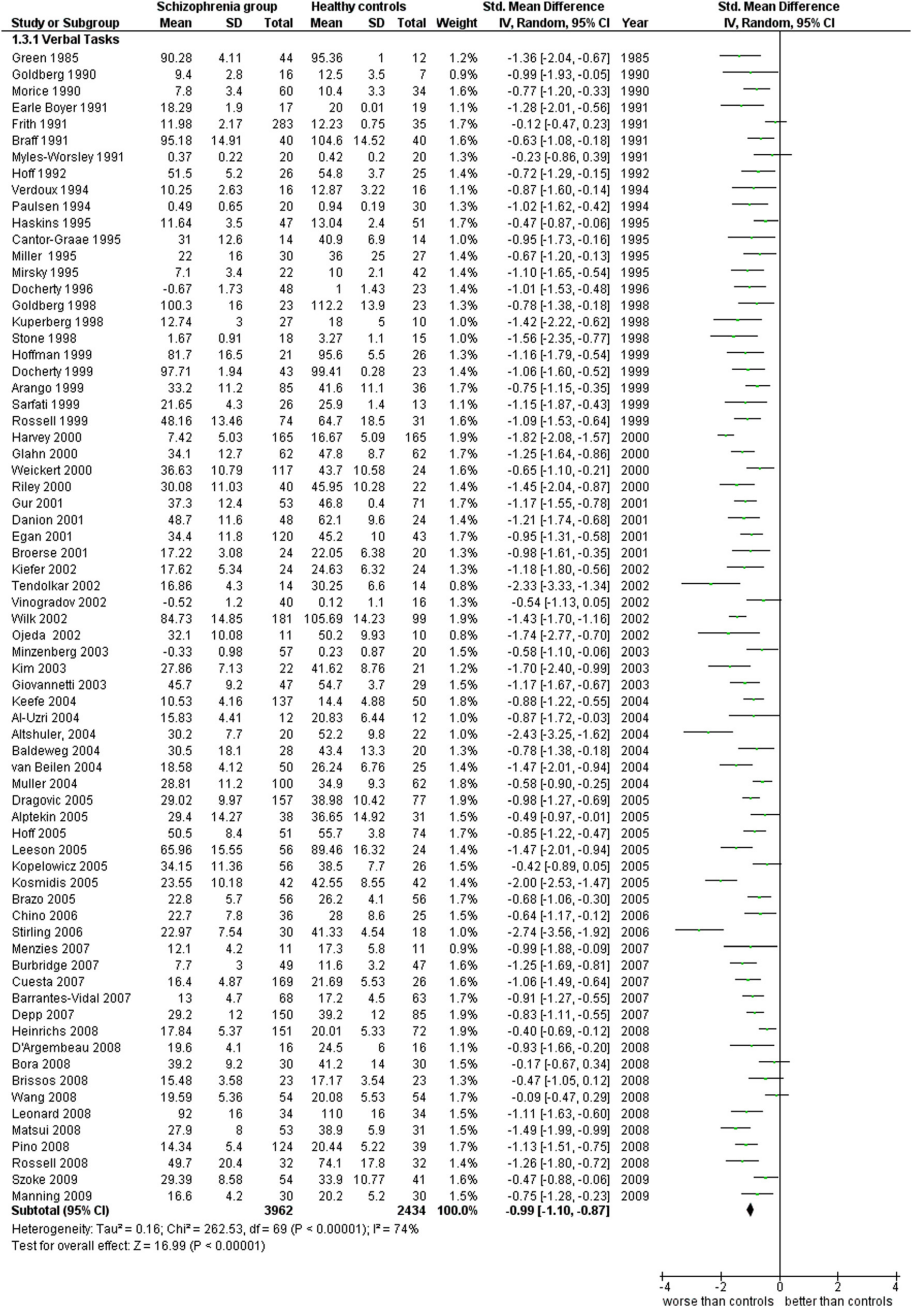
Verbal functioning.

Verbal functioning (70 studies). This analysis includes measures of fluency, naming tasks, etc. (see Additional file 1: Table S1 and Figure 12).

Executive Function

**Figure 13:**
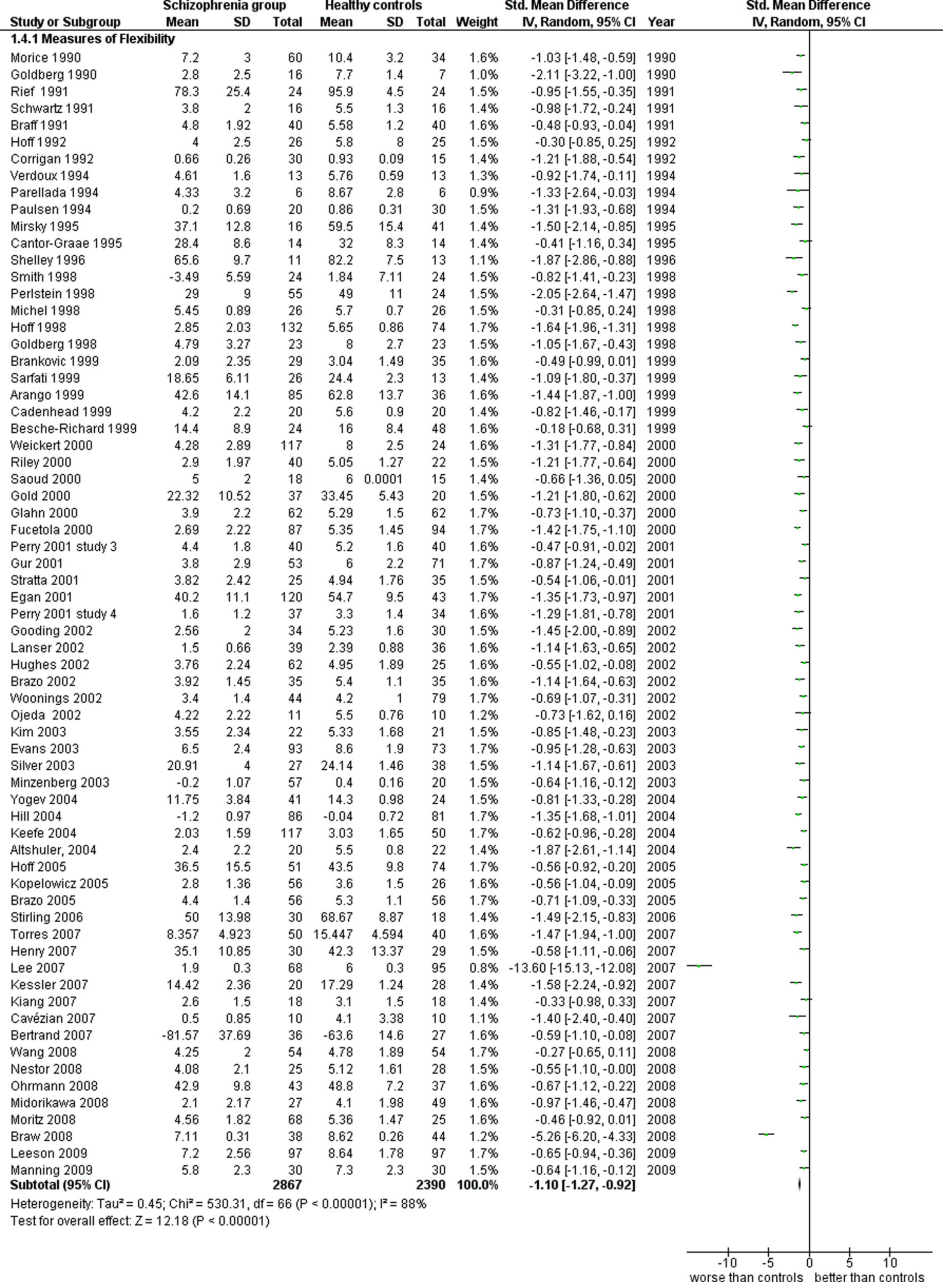
Measures of cognitive flexibility.

Measures of cognitive flexibility (67 studies). This analysis includes tasks principally obtained from the Wisconsin Card Sorting Test (see Additional file 1: Table S1 and Figure 13).

Attention

**Figure 14:**
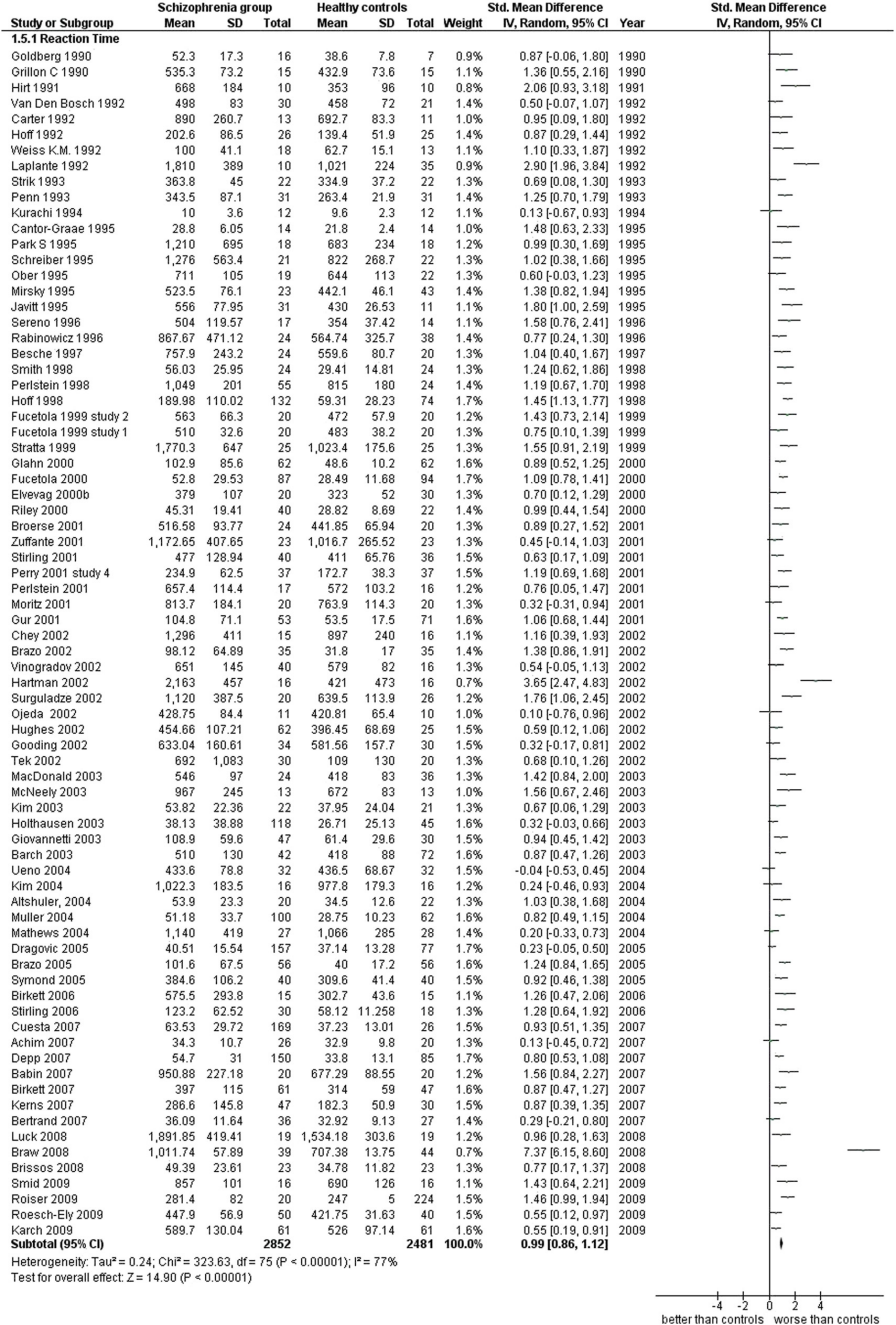
Reaction time.

**Figure 15:**
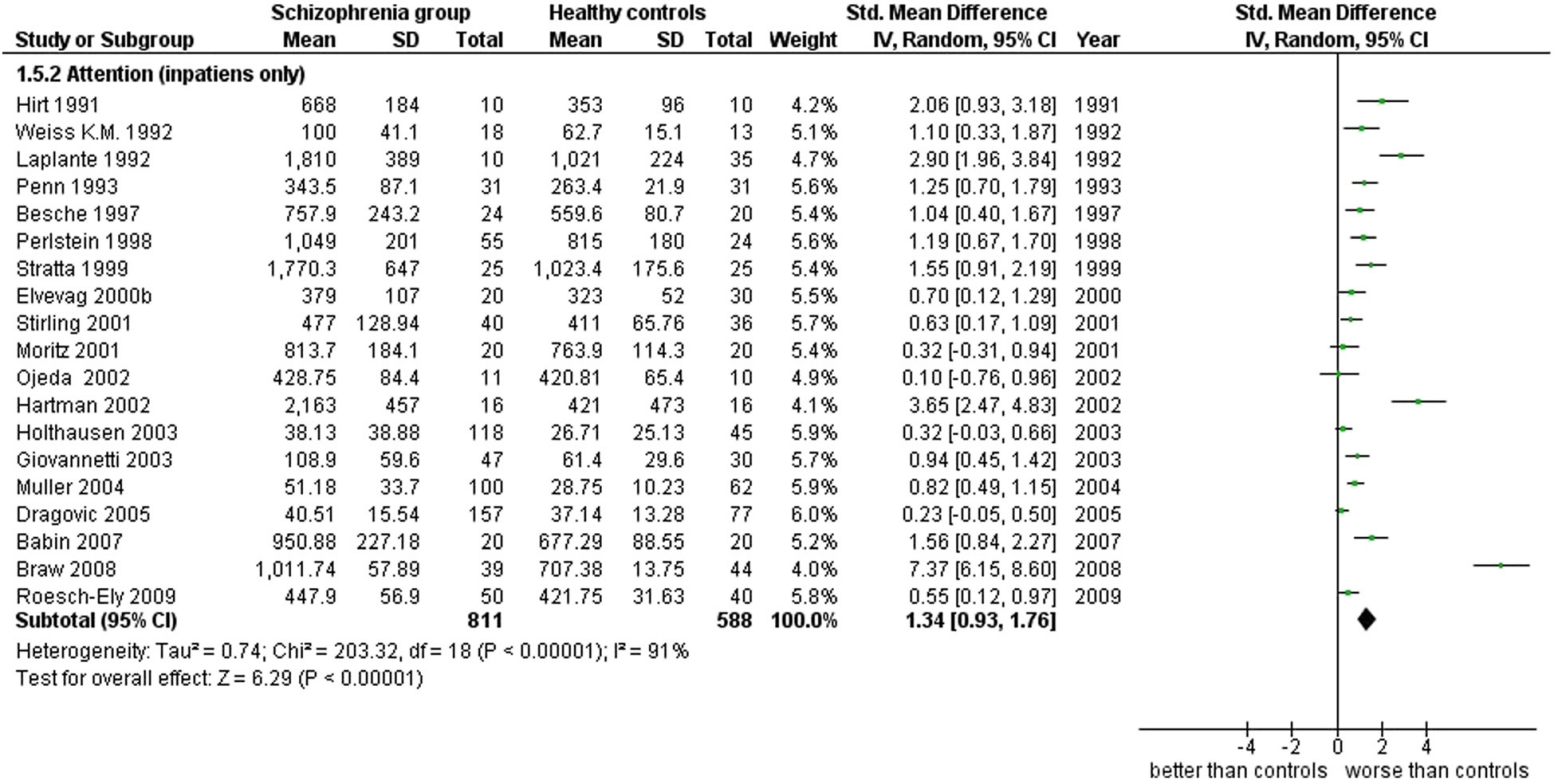
Attention (inpatients only).

**Figure 16:**
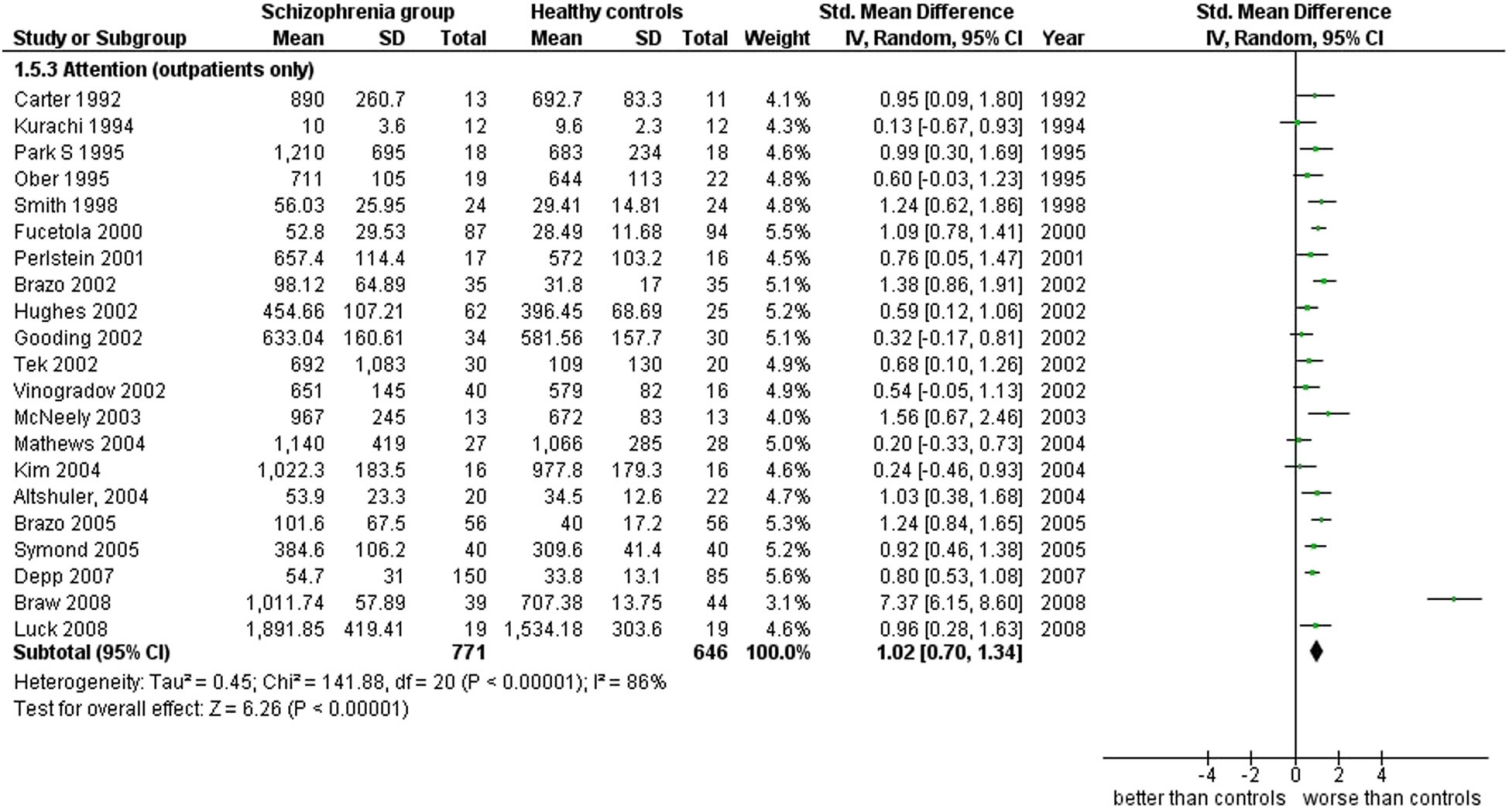
Attention (outpatients only).

Reaction Time (76 studies). This analysis includes the reaction time registered with various techniques and in various types of tasks (see Additional file 1: Table S1 and Figure 14).

Attention (inpatients only) (19 studies). This analysis includes only those studies where it was specified that the cases were inpatients (see Additional file 1: Table S1 and Figure 15).

Attention (outpatients only) (21 studies). This table includes only those studies where it was specified that the cases were outpatients (see Additional file 1: Table S1 and Figure 16).

**Table 1:** Distribution of the included studies in the different cognitive domains

** Measures of Memory Efficiency**	** Digit Span**
Altshuler (2004)^b^; Baldeweg (2004); Bora (2008)^b^; Braff (1991)^b^; Braw (2005)^a;b^; Brebion (2001) ^a^; Broerse (2001); Buckley (1994); Cadenhead (1999); Cantor-Graae (1995); Cavézian (2007); Clare (1993); Crespo- Facorro (1999)^a^; Egan (2001)^b^; Frith (1991); Goldberg (1990); Gras-Vincendon (1994); Hartman (2003)^a^; Henry (2007)^b^; Hill (2004)^a^; Hoff (1998); Holthausen (2003)^a^; Joyce (2002)^b^; Katz (2007); Kiefer (2002)^a^; Kopelowicz (2005)^a;b^; Kravariti (2003)^a^; Lanser (2002)^a^; Michel (1998); Minzenberg (2003)^b^; Mulholland (2008)^a^; Nestor (1998)^a^; Neufeld (1995)^a^; Niekawa (2007); Park S (1995)^b^; Perlstein (2001)^b^; Rodriguez-Sànchez (2007); Roesch-Ely (2009)^a^; Ross (2000)^b^; Silver (2003); Soriano (2009)^b^; Stone (1998)^a^; Stratta (1999 a)^a^; Tek (2002)^b^; Van Erp (2008)^b^; Weiss (2002)^b^; Woonings (2002)^a^.	Achim (2007); Alptekin (2005); Baldeweg (2004); Bertrand (2007); Birkett (2006); Brébion (2001)^a^; Brissos (2008); Buckley (1994); Burbridge (2007); Cohen (1999); Conklin (2002); Deep (2007); Fucetola (2000); Javitt (1997); Kiefer (2002)^a^; Kircker (2001); Kurachi (1994); Leonard (2008); Matsui (2008); Minzenberg (2003)^b^; Papageorgiou (2003); Rodriguez-Sànchez (2007); Roiser (2009); Schuepbach (2004); Silver (2003); Stirling (2006); Stone (1998)^a^; Thomas (1996); Waters (2006); Weiler (2009); Wood (2006).
	**LTM**
	Barrantes-Vidal (2007); Birkett (2006); Brazo (2002); Buckley (1994); Conklin (2002); Davidson (1996); Deep (2007); Dickinson (2004); Dragovic (2005); Evans (2003); Fucetola (2000); Gonzàlez-Blanch (2008); Green (1985); Gur (2001); Harvey (1988); Harvey (2000); Heinrichs (2008); Hill (2004b); Hoff (2005); Huges (2002); Kerns (2007); Kiang (2007); Kim (2003); Kravariti (2003); Leeson (2005); Leonard (2008); MacDonald (2003); Manning (2009); Menzies (2007); Michel (1998); Midorikawa (2008); Müller (2004); Nestor (1998); Ohrmann (2008); Pino (2008); Riley (2000); Schmand (1992); Schuepbach (2004); Seidman (2003); Skelley (2008); Tendolkar (2002); Van Beilen (2004); Wang (2008); Wilk (2002); Wood (2006).
**STM**	
Achim (2007); Arango (1999); Barch (2008); Barrantes-Vidal (2007); Birkett (2006); Brebion (2004); Brissos (2008); Cellard (2010); Chey (2002); Cosman (2009); Danion (2001); Dickinson (2004); Dragovic (2005); Fucetola (2000); Goldberg (1998); Gonzàlez-Blanch (2008); Gooding (2002); Gras-Vincendon (1994); Gur (2001); Harvey (1990); Hazlett (2000); Hill (2004b); Hoff (1992); Hoff (1998); Hoff (2005); Huddy (2009); Huges (2002); Keefe (2004); Kerns (2007); Kiang (2007); Kim (2003); Kopelowicz (2005); Landro (1993); Leeson (2005); Leonard (2008); MacDonald (2003); Manning (2009); Midorikawa (2008); Muller (2004); Nestor (1998); Ohrmann (2008); Palmer (2010); Pino (2008); Riley (2000); Schuepbach (2004); Seidman (2003); Skelley (2008); Snyder (2008); Tendolkar (2002); Van Den Bosch (1992); Verdoux (1995); Wang (2008); Weickert (2000); Wexler (1998); Wilk (2002); Wood (2006).	
	**Global Cognitive Functioning**
	Achim (2007); Aloia (1998) ^a^; Baas (2008); Badcock (2005); Badcock (2008); Barch (2008) ^b^; Barrantes-Vidal (2007) ^b^; Bell (2009); Bertrand (2007); Besche (1997)^a^; Bora (2008) ^b^; Braff (1991)^b^; Brazo (2002)^b^; Brazo (2005) ^b^; Brisson (2008); Burbridge (2007) ^b^; Cadenhead (1999); Cellard (2007)^a; b^; Cellard (2010); Chen (2008) ^b^; Conklin (2002) ^a^; Conklin (2005); Cuesta (2007); Danion (2001)^b^; Deep (2007) ^b^; Dickinson (2004) ^b^; Doughty (2008) ^a^; Edell (1987) ^a^; Egan (2001)^b^; Elvevag (2000)^a^; Elvevag (2000b)^a^; Elvevag (2001)^a^; Elvevag (2003); Frith (1991); Fucetola (2000) ^a^; Giovannetti (2003)^a^; Glahn (2000); Gold (2000) ^b^; Goldberg (1990); Goldstein (1998)^b^; Gooding (2002)^b^; Granholm (1991) ^b^; Granholm (1999); Gras-Vincendon (1994); Grillon ML (2010); Heaton (1994); Heinrichs (2008) ^b^; Henquet (2005) ^a^; Henry (2007) ^b^; Hoff (1992); Hoff (1998); Hoff (2005); Huddy (2009); Hughes (2002)^b^; Javitt (1995); Javitt (1997) ^a^; Keefe (2004); Kiehl (2005) ^b^; Krabbendam (2000)^b^; Kravariti (2003)^a; b^; Kurachi (1994); Langdon (2002); Lanser (2002)^a^; Leeson (2009); Leitman (2006); Leonard (2008); Luck (2008) ^b^; Macdonald (2003); Manning (2009) ^a^; Martin (2008); McNealy (2003) ^b^; Michel (1998) ^b^; Midorikawa (2008); Minzenberg (2003) ^b^; Morice (1990); Morrison-Stewart (1991); Mulholland (2008) ^a^; Nestor (2008); Neufeld (1978) ^a^; O’ Carroll (1999); Ohrmann (2008)^a^; Okada (2002)^a^; Park IH (2008)^b^; Penn (1993)^a^; Perry (Study 1) (2001)^a^; Perry (Study 3) (2001); Premkumar (2008)^b^; Rodriguez-Sànchez (2007); Ross (2000)^b^; Rund (2004)^a^; Sayers (1995)^a^; Schreiber (1995); Seidman (2003); Silver (2003); Skelley (2008); Stirling (2001)^a^; Stirling (2006); Sullivan (1994)^a^; Vinogradov (2002)^b^; Wang (2008)^a^; Weikert (2000); Zuffante (2001).
**Premorbid IQ**	
Altshuler (2004); Baas (2008); Badcock (2005); Badcock (2008); Baldeweg (2004); Birkett (2006); Brebion (2004); D’ Argembeau (2008); Doughty (2008) ^a^; Egan (2001)^b^; Elvevag (2000)^a^; Elvevag (2000b)^a^; Elvevag (2001)^a^; Elvevag (2003); Frith (1991); Goldberg (1998); Henry (2007) ^b^; Hill (2004); Hoff (1992); Horan (2009); Huddy (2009); Hughes (2002)^b^; Joyce (2002); Kiehl (2005) ^b^; Kircher (2001); Kuperberg (1998); Leeson (2009); Majoreck (2009); Manning (2009) ^a^; Menzies (2007); Moritz (2005); Moritz (2008); Phillips (2000); Roesch-Ely (2009); Roiser (2009); Rossell (1999); Rossell (2008); Schmand (1992); Smith (1998); Snyeder (2008); Stirling (2001)^a^; Stirling (2006); Sullivan (1994)^a^; Sullivan (2004); Surguladze (2002); Tsoi (2008); Waters (2006); Wood (2006).	
**Language**	
Alptekin (2005); Altshuler (2004); Al-Uzri (2004); Arango (1999)^b^; Baldeweg (2004); Barrantes-Vidal (2007); Bora (2008); Braff (1991)^b^; Brazo (2005); Brissos (2008); Broerse (2001); Burbridge (2007); Cantor-Graae (1995); Chino (2006); Cuesta (2007); D’ Argembeau (2008); Danion (2001); Deep (2007); Docherty (1996); Docherty (1999); Dragovic (2005); Earle Boyer (1991); Egan (2001)^b^; Frith (1991); Giovannetti (2003)^a^; Glahn (2000); Goldberg (1990); Goldberg (1998)^a^; Green (1985); Gur (2001); Harvey (1990); Haskins (1995); Heinrichs (2008); Hoff (2005); Hoff (1992); Hoffmann (1999); Keefe (2004); Kiefer (2002)^a^; Kim (2003); Kopelowicz (2005); Kosmidis (2005); Kuperberg (1998); Leeson (2005); Leonard (2008); Manning (2009); Matsui (2008); Menzies (2007); Miller (1995); Minzenberger (2003); Mirsky (1995); Morice (1990); Muller (2004); Myles-Worsley (1991); Ojeda (2002)^a^; Paulsen (1994); Pino (2008); Riley (2000); Rossell (1999); Rossell (2008); Sarfati (1999); Stirling (2006); Stone (1998)^a^; Szoke (2009); Tendolkar (2002); Van Beilen (2004); Verdoux (1995); Vinogradov (2002)^b^; Wang (2008); Weickert (2000); Wilk (2002).	
	**Executive Function**
	Altshuler (2004); Arango C. (1999)^b^; Bertrand (2007); Bersche-Richard (1999); Braff (1991)^b^; Brankovic (1999); Braw (2008); Brazo (2002)^b^; Brazo (2005); Cadenhead (1999); Cantor-Graae (1995); Cavèzian (2007); Corrigan (1991); Egan (2001)^b^; Fucetola (2000); Glahn (2000); Gold (2000); Goldberg (1990); Goldberg (1998)^a^; Gooding (2002)^b^; Gur (2001); Henry (2007) ^b^; Hill (2004); Hoff (1992); Hoff (1998); Hoff (2005); Hughes (2002)^b^; Keefe (2004); Kesserl (2007); Kiang (2007); Kim (2003); Kopelowicz (2005); Lanser (2002)^a^; Lee (2007); Leeson (2009); Manning (2009) ^a^; Michel (1998); Midorikawa (2008); Minzenberg (2003); Mirsky (1995); Morice (1990); Moritz (2008); Nestor (2008); Ohrmann (2008); Ojeda (2002)^a^; Parellada (1994); Paulsen (1994); Perlstein (1998)^a^; Perry (Study 3) (2001); Perry (Study 4) (2001); Rief (1991); Riley (2000); Saoud (2000); Sarfati (1999); Schwartz (1991); Shelley (1996); Silver (2003); Smith (1998); Stirling (2006); Stratta (2001); Torres (2007); Verdoux (1995); Wang (2008); Weickert (2000); Woonings (2002)^a^; Yogev (2004).
**Attention**	
Achim (2007); Altshuler (2004) ^b^; Babin (2007) ^a^; Barch (2003); Bertrand (2007); Besche (1997)^a^; Birkett (2006); Birkett (2007); Braw (2008) ^a; b^; Brazo (2002)^b^; Brazo (2005) ^b^; Brisson (2008); Broerse (2001); Cantor- Graae (1995); Carter (1992)^b^; Chey (2002); Cuesta (2007); Deep (2007) ^b^; Dragovic (2005) ^a^; Elvevag (2000b); Fucetola (study 1) (1999); Fucetola (study 2) (1999); Fucetola (2000) ^b^; Giovannetti (2003)^a^; Glahn (2000); Goldberg (1990); Gooding (2002)^b^; Grillon C (1990); Grillon ML (2010); Gur (2001); Hartman (2003)^a^; Hirt (1991)^a^; Hoff (1992); Hoff (1998); Holthausen (2003) ^a^; Hughes (2002)^b^; Javitt (1995); Karch (2009); Kerns (2007); Kim (2003); Kim (2004) ^b^; Kurachi (1994)^b^; Laplante (1992)^a^; Luck (2008)^b^; MacDonald (2003); Mathews (2004) ^b^; McNealy (2003) ^b^; Mirsky (1995); Moritz (2001)^a^; Muller (2004) ^a^; Ober (1995)^b^; Ojeda (2002)^a^; Park S (1995)^b^; Penn (1993)^a^; Perlstein (1998)^a^; Perlstein (2001)^b^; Perry (Study 4) (2001)^a^; Rabinowicz (1996); Riley (2000); Roesch-Ely (2009) ^a^; Roiser (2009); Schreiber (1995); Sereno (1996); Smid (2009); Smith (1998) ^b^; Stirling (2001)^a^; Stirling (2006); Stratta (1999)^a^; Strik (1993); Surguladze (2002); Symond (2002) ^b^; Tek (2002)^b^; Ueno (2004); Van Den Bosh (1992); Vinogradov (2002)^b^; Weiss (1992)^a^; Zuffante (2001).	

^a^ : studies with cases as inpatients; ^b^ : studies with cases as outpatients.

In general, since there isn’t any substantial change with respect to the measures utilized in the previous review, the same detailed descriptive table of the measures found in the previous review is valid also for this updated version [7].

### Description of the studies

The clinical criteria to individuate and select the patients of the studies added to this review are the same as those of the previous version (in general they were defined according to DSM III, DSM III R, DSM IV, DSM IV-TR, ICD 9, ICD 10, and RCD the Research Diagnostic Criteria). Only in few instances there was a distinction of patients according to different types of diagnosis, but since this information is present in very few papers, it has not been utilized for this review. The total number of cases of this updated version of the review is 18,049: 10,120 patients of the Schizophrenia Group (SG) and 7,929 normal cases of the Control Group (CG).

As shown in Table 2, the unbalance between the number of patients and that of the normal cases is persistent and generalized in all analyses but one. It ranges between a maximum of 38% of patient surplus with respect to the normal cases to a minimum of 12%. Only the analysis concerning the IQ measures has a balanced number of patients and normal cases. The systematic unbalance between the groups was already identified in the previous review and it has remained unchanged in the more recent papers. This unbalanced design is not due to few big studies where there was a an asymmetrical recruitment of patients and controls, but it is due to a generalized and persistent modality of recruitment of the great majority of studies.

**Table 2:** Summary of results

** Outcome**	**N Studies**	**N Total cases**	**N SG**	**N CG**	CG/SG*100	**Effect Size C.I. 95%**	**PS**	**I**^**2**^
*1 Memory functioning*	*128*							
1.1 Measures of Memory Efficiency	47	3,432	2,066	1,366	66.12	−1.22 [-1.44, -1.01]*	0.81	86%*
1.2 Measures of Memory Functioning (inpatients only)	17	1,183	630	553	87.78	−1.21 [-1.63, -0.80]*	0.80	90%*
1.3 Measures of Memory Functioning (outpatients only)	16	1,162	678	484	71.39	−1.83 [-2.35, -1.31]*	0.90	92%*
1.4 Digit Span	31	2,092	1,209	883	73.04	−0.67 [-0.81, -0.53]*	0.68	51% **
1.5 LTM	45	5,045	2,801	2,244	80.11	−1.14 [-1.32, -0.96]*	0.79	87%*
1.6 STM	56	5,405	3,032	2,373	78.26	−1.05 [-1.18, -0.92]*	0.77	77%*
*2 Global cognitive functioning*	*131*							
2.1 Measures of IQ	102	8,416	4,760	3,656	76.81	−0.96 [-1.07, -0.85]*	0.75	80%*
2.2 Measures of IQ (inpatients only)	27	1,800	894	906	101.34	−1.04 [-1.25, -0.82]*	0.77	77%*
2.3 Measures of IQ (outpatients only)	27	2,274	1,355	919	67.82	−0.83 [-1.00, -0.66]*	0.72	69%*
2.4 premorbid IQ	48	3,568	2,049	1,519	74.13	−0.57 [-0.70, -0.43]*	0.65	70%*
*3 Language*	*70*							
3.1 Verbal Tasks	70	6,396	3,962	2,434	61.43	−0.99 [-1.10, -0.87]*	0.76	74%*
*4 Executive function*	*67*							
4.1 Measures of Flexibility	67	5,257	2,867	2,390	83.36	−1.10 [-1.27, -0.92]*	0.78	88%*
*5 Attention*	*76*							
5.1 Reaction Time	76	5,333	2,852	2,481	86.99	0.99 [0.86, 1.12]*	0.76	77%*
5.2 Attention (inpatients only)	19	1,399	811	588	72.50	1.34 [0.93, 1.76]*	0.83	91%*
5.3 Attention (outpatients only)	21	1,417	771	646	83.79	1.02 [0.70, 1.34]*	0.76	86%*

SG = Patients with Schizophrenia Group; CG normal control cases group.

CG/SG*100: percentage of control cases with respect of patients.

PS = probability of superiority; I^2^ = percentage of heterogeneity.

* p < .00001; ** p < .00008.

The mean age of cases described in the single studies ranges between a minimum of 16.5 years for patients of the SG and of 16.2 years of the CG to a maximum of 73.3 years in both groups.

Not all the studies describe the composition of their cases and patients by sex. When this information is available, the mean percentage of males is 70.60% in the SG and 60.81% in the CG with a range from a minimum of 0% (all females) to a maximum of 100% (all males). The average number of male cases of the CG is 87% of the average number of male patients of the SG.

In general, most of the patients were examined while taking antipsychotic drugs but it is very rare to find specified the length of the therapeutic treatment or the eventual suspension of it in occasion of the cognitive evaluation. In few instances, it is indicated that therapies different from antipsychotic drugs were also administered to the patients such as BZD, antidepressants, etc.

Many studies do not specify if patients were examined in an acute, chronic, or remission phase.

The inclusion of data in specific analyses was decided according to the description offered by the authors of the single papers.

### Data analysis

All analyses are performed comparing patients with schizophrenia to normal cases using Review Manager (RevMan) Version 5.1., Copenhagen: The Nordic Cochrane Centre, The Cochrane Collaboration, 2011. All variables used are continuous measures which are analyzed by the Standardized Mean Difference, Random Effect Models, due to diversity of methods of measurement used in each analysis, to the randomness of patients sampling empirically done in each of the included studies, and to the high level of heterogeneity of their variance. The heterogeneity is also quantified by the index I^2^[8] which indicates the part of variance due to the presence of specific causes different from chance but not equally distributed in all the studies considered. In those instances where the original data were presented as different subgroups of patients, these data were recomputed in order to be inserted as a single group of patients.

The type of studies included in this meta-analysis does not require a quality assessment of the randomization procedure of allocation of cases. It has other quality assurance requirements, mostly devoted to warrant a sound methodological quality of results. The quality analysis was carried out adopting the method proposed by Egger et al. [9] which evaluates the presence of interfering factors on the results by the method of meta-regression. The meta-regression was used to investigate the relationship of sex, age, and number of participants with the magnitude of the effect size of the single cognitive areas.

Finally, the effect size of each cognitive function was transformed according to the method proposed by Grissom [10] to the probability of superiority estimate (PS index) which allows for the quantification of the probability [11] that a case from the schizophrenia group will present a score different from that obtained by a case from the control group for each of the cognitive areas examined.

## Results and discussion

### Memory functioning

In this cognitive area concerning the measures of memory functioning, the comparison between 2,066 patients with schizophrenia and 1,366 normal subjects (47 studies for a total of 3,432 cases) produces an ES = −1.22 [-1.44, -1.01] with an I^2^ = 86% and a PS = 81%. These results demonstrate that there is a significant decline of memory functioning among the patients with schizophrenia confirmed by the high probability (81%) to find a patient with a memory impairment vs. a 19% of probability to find a patient with scores similar to those of a normal case. The high heterogeneity of these studies limits the usefulness of these findings, since it is not possible to exclude that factors other than the diagnosis could contribute, at least partially, to determine the difference between patients and normal subjects (see Table 2).

The results obtained for the same area, separating inpatients from outpatients, apparently show that outpatients have a larger difference from normal subjects but also maintain a very large amount of heterogeneity between studies (respectively: inpatients, ES = −1.21 [-1.63, -0.80], I^2^ = 90%, PS = 80%; outpatients, ES = −1.83 [-2.35, -1.31], I^2^ = 92%, PS = 90%). The number of studies was similar in both instances. In this analysis, the difference of ES magnitude between in and out-patients is only apparent. In fact, their confidence intervals overlap in a way that let us exclude that the two ES’s can be considered different.

An example of the influence of the methodological heterogeneity on the ES is offered by the results concerning data obtained by the systematic use of a single type of measure in all studies on memory functioning; in this case the Digit Span. In a total of 2,092 cases from 31 works, there is an ES = −0.67 [-0.81, -0.53] with an I^2^ 51%. These results show that when a source of variance due to the differences between measurement methods employed in the different studies is eliminated, there is a consistent reduction of the effect size (which is still demonstrative of a statistically significant difference between groups) accompanied by a reduction to almost half of the heterogeneity.

Analysis of data in function of the type of memory model adopted in the studies was carried out in order to control for a likely source of heterogeneity. The data allowed us to separate results concerning short term memory (STM) vs. those concerning long term memory (LTM). Other models of memory were not suited for this type of analysis since specific data were only sporadically available.

When only LTM data are included in the analysis, from 45 studies for a total of 5,045 cases, ES is −1.14 [-1.32, -0.96], I^2^ 87%, and PS 79%. Similar but slightly less intense results are obtained for STM data, obtained from 56 studies for a total of 5,405 cases, where ES = −1.05 [-1.18, -0.92], I^2^ 77%, PS 77%.

These results show that the separation of the type of memory model reduces the heterogeneity, while the use of only a specific memory measure cuts the heterogeneity to a much less amount (from around 90% when put together to about 80% when separated by memory model and to 50% when using a single type of measure). In synthesis, these results show that, without a question, there is a reduction in memory functioning among the patients with schizophrenia, whatever the method of examination. They also demonstrate that, at least for memory functioning, it would be preferable, in the future research activity, to identify a specific method of measurement to be adopted on the basis of research hypotheses and feasibility of use in this clinical area, with respect to the current practice of evaluating memory functioning with whatever task is occasionally available at the moment. The heterogeneity would be greatly reduced and the results would be much more informative.

### Global cognitive functioning

This area was evaluated in general, by IQ measures (102 works, 8,416 total cases). The ES is −0.96 [-1.07, -0.85] with I^2^ = 80% and PS = 75%. When outpatients’ and inpatients’ results are separated and analyzed, the former group has an ES = −0.83 [-1.00, -0.66] I^2^ 69%, PS 72% while the latter group has an ES = −1.04 [-1.25, -0.82], I^2^ 77%, PS 77%. These results show how the cognitive impairment is generic and diffuse among the patients with schizophrenia, in at least 3 patients out of 4, and it is not dependent on the severity of the disease (inpatients and outpatients do not differ very much in their results concerning the IQ).

The data concerning the premorbid IQ, are in general measured by NART or WRAT or using specific subtests of the WAIS considered stable over-time. These data are based on 48 works for a total number of 3,568 cases and show an ES = −0.57 [-0.70, -0.43], I^2^ = 70%, PS 65 = %. The hypothesis based on the premorbid IQ, that some cognitive discrepancies are already present in the patients population years ahead of an explicit expression of the clinical features of this disease, might be confirmed by these results, at least in 2 cases every 3. Naturally, since the largest part of these pre-morbid data are retroactively reconstructed when the disease is already diagnosed, it seems necessary a further confirmation of this hypothesis by the longitudinal proactive method of study where the pre-morbid IQ data are obtained before the diagnosis.

In general, IQ data confirm the findings already seen for the memory functioning both in terms of ES and of a large heterogeneity. It must be noted that the heterogeneity is around 80% when an homogeneous function is evaluated, such as the IQ or specific models of memory functioning,

### Language

The language functioning was evaluated in 70 works for a total of 6,396 cases (3,962 SG and 2,434 CG). The ES is −0.99 [-1.10, -0.87] with I^2^ = 74% and PS = 76%.

### Executive function

Data concerning this cognitive area were studied in 67 works for a total of 5,257 cases (2,867 SG and 2,390 CG). The ES is −1.10 [-1.27, -0.92] with I^2^ = 88% and PS = 78%.

Both measures of language functioning and executive function show that SG patients obtain significantly worse results than those obtained by the normal controls. The magnitude of differences is similar to that of the other areas already examined and the same happens for the heterogeneity.

### Attention

Data in this cognitive area are measures of reaction time, obtained in a variety of techniques and tasks from 76 studies for a total of 5,333 cases (2,852 SG and 2,481 CG). The ES is 0.99 [0.86, 1.12] with I^2^ = 77% and PS = 76%. When inpatients are separately analyzed from outpatients, the inpatients’ ES is 1.34 [0.93, 1.76] with I^2^ = 91% and PS = 83%, while the outpatients’ ES is 1.02 [0.70, 1.34] with I^2^ = 86% and PS = 76%. Patients with schizophrenia have a slower reactivity to stimuli than normal cases and in particular there is a slight stronger tendency of this to happen among the inpatients (4 out of 5 inpatients are probably found slower in their RT’s with respect to 3 out of 4 outpatients).

### Meta-regression

The ES for every type of analysis was correlated with number of cases, sex distribution, and age of participants of each group in order to identify the role of these structural variables in the identification of the between group differences expressed in terms of ES.

The number of cases has a significant effect on the between group differences for the pre-morbid IQ, the memory functioning (outpatients only), and the attention measures (expressed in terms of reaction time). We must remind that there is a wide and generalized imbalance between group composition for all cognitive variables examined in this meta-analysis (Table 2).

The composition by sex of the groups has a significant effect on the between group difference for the IQ measures, the memory functioning, the language functioning, the executive function, and the attention measures. In general, there is an unbalance of sex composition between the SG and CG groups (Table 2).

The age of participants has a significant effect on between group differences for the executive function and the attention measures.

In particular, the between group difference on measures of pre-morbid IQ seems to be partially related to the unbalance of number of cases in the two groups (respectively increasing the number of SG patients decreases the ES, p < .04, while increasing the number of CG cases increases it, p < .04). The magnitude of the ES concerning the IQ measures seems to be related to the differences in sex distribution, in particular for the SG group (p < .003) where increasing the number of females reduces the difference between SG and CG groups. The memory functioning measures (outpatients only) show that increasing the number of patients of the SG group and the number of male cases of the CG decreases the between group difference. For the language functioning measures the increment of males in the CG increases the between group difference (p < .05). For the executive function measures the increasing of age of the CG (p < .03) and the number of females patients of the SG (p < .04) decreases the between group difference. The measure of RT’s of inpatients shows that increasing the number of the SG patients decreases the between group difference (p < .04). The measures of RT’s in general show that an increase of number of males of the CG (p < .001) and the age of SG patients (p < .001) increases the between group difference.

All these results show that the reduction of the discrepancies and the unbalance of composition of the groups, together with the reduction of the heterogeneity, could produce a parallel reduction of the magnitude of the ES. For what is possible to see from our analysis, despite this attenuation of effects, the present differences would remain significant in most domains.

## Conclusions

This updated version of the meta-analysis on cognitive deficits of patients with schizophrenia evidenced by the comparison with normal control cases, has confirmed the stability of the results found in the previous work [7]. These findings show a generalized presence of cognitive impairment among the patients with schizophrenia. These results cannot be considered free of the potential bias that only controlled studies with positive results are available in the published evidence, while all those with negative results are not traceable. The real possibility of such a bias, should make us consider that the results obtained in this meta-analysis might be, in some degree, inflated by an underrepresentation of negative results.

Another problem is evidenced by the quality analysis of the included studies. The methodological characteristics of the studies on this cognitive impairment, could be improved with a better control on the balance of number of cases, sex composition of the two groups, and, at a less extent, age of participants. The balance of these factors will take care of some of the structural dysfunctional characteristics evidenced in this meta-analysis.

The identification of precise and replicable measurement procedures is another of the requirements that have demonstrated to be useful in reducing the methodological heterogeneity of the present results. By means of the standardization of methodology, the studies on cognitive deficits of patients with schizophrenia might move, from the current situation where they are mostly descriptive, to the level where they could be of help in refining and confirming explanatory hypotheses concerning the characteristics and the nature of the cognitive impairment.

In the course of the updating process of this meta-analysis, various characteristics of the available data have come to attention. It is of general knowledge that there is a reduction of cognitive efficiency in patients with schizophrenia, but it is important to consider that the intensity of this reduction (evidenced by comparing patients’ results to those of normal cases) is not sufficient to classify most of the patients’ level of functioning below the normal limits. As an example, the range of mean IQ level, found in the studies concerning patients is 84–107 which indicates that in general, the average intellectual abilities of the groups of patients studied are not below the medium-low level of classification. Analogous considerations could be made about the memory efficiency and the other cognitive areas explored, based on the magnitude of the effect sizes obtained in the analyses.

All the elements evidenced in the discussion, converge on the high heterogeneity found among the studies. A heterogeneity so high as that found in our results, shows that there are diffuse and structural problems in considering all the studies performed in this area of research as belonging to the same class of studies. It is necessary to reduce the heterogeneity to the only acceptable source in this area of research, the clinical heterogeneity, dependent on clinical and functional differences among patients classified in the same diagnostic area. In order to obtain this simplification of heterogeneity, it is necessary to eliminate or control for the other unwanted sources of heterogeneity, principally the methodological heterogeneity. This could be obtained by developing and adopting a standardized and consensus-based set of measurement procedures and criteria for identification and selection of cases for the groups to be studied. It is possible to foresee from our results, that improving the methodological models adopted for each study, there will be a reduction of the heterogeneity and an attenuation of the differences commonly found between patients and normal subjects in most of the cognitive domains. It seems likely from our results that even if attenuated, in most cases, these differences will remain statistically significant.

## Competing interests

The authors declare that they have no competing interests.

## Authors’ contributions

MF monitored the data base search and papers selection. Executed the statistical analyses and contributed to the preparation of the manuscript. VB carried out the data base search and the initial selection of the to be included papers. Contributed to the data extraction from the single papers. MEC carried out the data base search and the phases of selection of the to be included papers. Executed the data extraction and input process. Contributed to the preparation of the manuscript. All authors read and approved the final manuscript.

## Authors’ information

MF: Professor of Clinical Psychology, Member of the Editorial Board of the Cochrane Dementia and Cognitive Improvement Group, Oxford, UK.

VB: Clinical Psychologist and Psychotherapist.

MEC: Clinical Psychologist and Psychotherapist, author of other metanalyses.

## Pre-publication history

The pre-publication history for this paper can be accessed here:

http://www.biomedcentral.com/1471-244X/12/64/prepub

## Supplementary Material

Additional file 1 **Table S1.** Summary of included papers. Click here for file
