# Supplementary material for: Cognitive deficits in schizophrenia: an updated metanalysis of the scientific evidence
Source: BMC Psychiatry. 2012 Jun 20;12:64. doi: 10.1186/1471-244X-12-64 (PMC3528440; doi:10.1186/1471-244X-12-64)
Supplement: Additional file 1 — Table S1. Summary of included papers. [file 1471-244X-12-64-S1.doc]

*Tab. 2 – Summary of included papers*

| **ID** | **Memory** | **IQ** | **Language** | **Executive Functioning** | **Attention** |
| --- | --- | --- | --- | --- | --- |
| Achim 2007 | Digit Span; WMS logical memory (STM) | WAIS III |  |  | TMT-A |
| Aloia 1998 |  | WAIS |  |  |  |
| Alptekin 2005 | Digit Span |  | COWAT |  |  |
| Altshuler 2004 | CVLT total score | premorbid ANART | FAS | WCST (categories) | Trail A |
| Al-Uzri 2004 |  |  | categories Fluency |  |  |
| Arango 1999 | WMS logical memory |  | Verbal Fluency | Category fluency |  |
| Baas 2008 |  | Premorbid NART; Matrices |  |  |  |
| Babin 2007 |  |  |  |  | RT |
| Badcock 2005 |  | SILS; NART IQ |  |  |  |
| Badcock 2008 |  | Premorbid NART; Shipley IQ |  |  |  |
| Baldeweg 2004 | Digit span; RBMT tot score | Premorbid NART | Verbal Fluency |  |  |
| Barch 2003 |  |  |  |  | AX-CPT (AX short delay) |
| Barch 2008 | n-back (0-back accuracy) | WAIS III Vocabulary |  |  |  |
| Barrantes-Vidal 2007 | WMS immediate and delayed | IQ estimate | COWAT |  |  |
| Bell 2009 |  | WASI |  |  |  |
| Bersche-Richard 1999 |  |  |  | Task of French category table inclusion (sentence generation) |  |
| Bertrand 2007 | Digit span | WAIS III |  | Trail B | Trail A |
| Besche 1997 |  | WAIS (vocabulary) |  |  | RT |
| Birkett 2006 | Digit span; Prose recall-immediate and delayed | Spot the word (premorbid verbal intelligence) |  |  | RT |
| Birkett 2007 |  |  |  |  | RT |
| Bora 2008 | ACT score | WAIS information | Verbal fluency |  |  |
| Braff 1991 | Memory (TPT) | WAIS-R (Verbal) | Verbal IQ (WAIS-R) | WCST (categories Achieved) |  |
| Brankovic 1999 |  |  |  | Reasoning task |  |
| Braw 2008 | SRM |  |  | SOC (problem solved) | Response latency (ms) |
| Brazo 2002 | CVLT | WAIS-R |  | WCST (categories) | TMT (B-A) |
| Brazo 2005 |  | WAIS | Verbal Fluency | MCST categories | TMT A |
| Brebion 2001 | Global Memory Efficiency; digit span and immediate recall |  |  |  |  |
| Brébion 2004 | Recalled words nonorganizable | NART |  |  |  |
| Brissos 2008 | Digit Span; WMS logical memory | WAIS-R Information | Verbal fluency |  | TMT A |
| Broerse 2001 | Rey Complex Figure |  | Verbal Fluency |  | CPT |
| Buckley 1994 | WMS-R General Memory; digit span; delayed recall |  |  |  |  |
| Burbridge 2007 | Digit span | WRAT 3 | CVLT |  |  |
| Cadenhead 1999 | Recognition Memory Test | WAIS-R; Vocabulary |  | WCST-categories |  |
| Cantor-Graae 1995 | Verbal Memory test |  | Vocabulary | Block Design | a reaction time test |
| Carter 1992 |  |  |  |  | Stroop Color Naming Task (neutral word) |
| Cavézian 2007 | Visual Memory |  |  | MVPT-V total score |  |
| Cellard 2007 |  | WAIS III |  |  |  |
| Cellard 2010 | 7 TBR items control | WAIS-R |  |  |  |
| Chen 2008 |  | Verbal IQ estimated |  |  |  |
| Chey 2002 | Spatial Delay Reponse (immediate) |  |  |  | RT |
| Chino 2006 |  |  | Letter Fluency Test |  |  |
| Clare 1993 | Warrington’s Recognition Memory Test |  |  |  |  |
| Cohen 1999 | Short Term Memory; word span |  |  |  |  |
| Conklin 2002 | RAVLT delay | IQ (prorated) |  |  |  |
| Conklin 2005 | Digit span | Block design+information |  |  |  |
| Corrigan 1992 |  |  |  | Component Recognition |  |
| Cosman 2009 | Word list memory (WM) |  |  |  |  |
| Crespo-Facorro 1999 | RAVLT |  |  |  |  |
| Cuesta 2007 |  | Information WAIS | Word fluency |  | TMT-A |
| D’Argembeau 2008 |  | French NART | Phonemic verbal fluency |  |  |
| Danion 2001 |  | WAIS | Verbal Fluency |  |  |
| Davidson 1996 | Verbal serial learning delayed and immediate recall |  |  |  |  |
| Depp 2007 | Digit Span; CVLT long delay free | WAIS vocabulary | Letter fluency |  | Trail A |
| Dickinson 2004 | WMS Logical memory (Immediate and Delayed recall) | WAIS full scale |  |  |  |
| Docherty 1996 |  |  | Boston Naming Test |  |  |
| Docherty 1999 |  |  | Communication Disturbances Index |  |  |
| Doughty 2008 |  | WASI; NART premorbid |  |  |  |
| Dragovic 2005 | RAVLT immediate and delayed |  | Verbal fluency |  | Inspection time |
| Earle Boyer 1991 |  |  | CPT-stimuli, Visual task lexical stimuli |  |  |
| Edell 1987 |  | WAIS (comprehension; similarities; vocabulary) |  |  |  |
| Egan 2001 | CVLT (Declarative Memory and delayed) | IQ; WRAT | Language production/retrieval | Working Memory (WCST-CAT) |  |
| Elvevag 2000 |  | WAIS; WRAT premorbid |  |  |  |
| Elvevag 2000b |  | Culture Fair IQ; NART IQ |  |  | Cued Spatial Location Task |
| Elvevag 2001 |  | WAIS; WRAT |  |  |  |
| Elvevag 2003 |  | WAIS-R; WRAT |  |  |  |
| Evans 2003 | Delayed recall |  |  | Abstraction/flexibility |  |
| Frith 1991 | forced-choise recognition memory for single words | Quick Test for IQ; NART | verbal fluency task (odd responses) |  |  |
| Fucetola 1999 (study 1) |  |  |  |  | Visual CPT (reaction time) |
| Fucetola 1999 (study 2) |  |  |  |  | Visual CPT (reaction time) |
| Fucetola 2000 | WAIS digit span; immediate and delayed | WAIS (Vocabulary and Block Design) |  | WCST (categories) | TMT Trail A |
| Giovannetti 2003 |  | WAIS-R | Boston Naming Test |  | TMT |
| Glahn 2000 |  | WAIS-R (Vocabulary) | Controlled Oral Word Association Task | WCST (categories) | TMT |
| Gold 2000 |  | WRAT |  | CAL correct responses on the unique task |  |
| Goldberg 1990 | WMS (Memory Quotient) | WAIS-R | Vocabulary | WCST (categories) | CPT Stimuli (Reaction Time) |
| Goldberg 1998 | WMS immediate recall | WRAT | Peabody Picture Vocabulary Test | WCST (categories) |  |
| Goldstein 1998 |  | WRAT-R |  |  |  |
| Gonzalez-Blanch 2008 | RBMT immediate and delayed |  |  |  |  |
| Gooding 2002 | Working memory (percent correct) | WAIS –R |  | WCST (categories) | RT |
| Granholm 1991 |  | WAIS-R vocabulary |  |  |  |
| Granholm 1999 |  | WAIS-R vocabulary |  |  |  |
| Gras-Vicendon 1994 | WMS; Free recall (number of words) | WAIS-R |  |  |  |
| Green 1985 | Bushke recall (LTM) |  | Token test |  |  |
| Grillon C 1990 |  |  |  |  | Auditory reaction time task (Distraction Condition) |
| Grillon ML 2010 |  | WAIS-R |  |  |  |
| Gur 2001 | Logical memory delayed recall; and immediate |  | Language ability (LAN) | WCTS (categories) | Trail B |
| Hartman 2002 | Memory (Cognistat) |  |  |  | DMTS |
| Harvey 1988 | Serial recall task |  |  |  |  |
| Harvey 1990 | Word Span Encoding Task (Neutral; Lenght 8 words) |  |  |  |  |
| Harvey 2000 | CERAD (delayed recall score) |  | CERAD (animal naming fluency) |  |  |
| Haskins 1995 |  |  | Emotional Blunting Scale |  |  |
| Hazlett 2000 | SVLT (number of correct recalls) |  |  |  |  |
| Heaton 1994 |  | WAIS-R |  |  |  |
| Heinrichs 2008 | CVLT II recall (LTM) | WAIS III vocabulary | COWAT semantic |  |  |
| Henquet 2005 |  | GIT |  |  |  |
| Henry 2007 | Verbal learning | WASI; NART premorbid |  | Phonemic Fluency |  |
| Hill 2004 | Verbal memory | WRAT premorbid |  | z scores of more test |  |
| Hill 2004 b | Short delay and long delay free recall |  |  |  |  |
| Hirt 1991 |  |  |  |  | Reaction Times at a visual task |
| Hoff 1992 | Logical Memory | Raven IQ; WRAT | Boston Naming Test | WCST(categories) | Cancellation Test |
| Hoff 1998 | Associate Learning and immediate memory | Verbal IQ |  | WCST (categories) | Concentration and Speed Test |
| Hoff 2005 | WMS immediate and delayed | Verbal IQ | Boston Naming test | Controlled oral word association |  |
| Hoffman 1999 |  |  | Continuous Performance Task |  |  |
| Holthausen 2003 | CVLT trial 1-5 |  |  |  | TMT A |
| Horan 2009 |  | WTAR |  |  |  |
| Huddy 2009 | Spatial Span (STM) | WTAR premorbid; WAIS III |  |  |  |
| Huges 2002 | Delayed Verbal Memory and immediate | IQ (full scale); NART |  | WCST (categories) | CPT reaction time |
| Javitt 1995 |  | Quick Test |  |  | Auditory Mean CPT (Reaction Time) |
| Javitt 1997 | Digit span | Quick Test |  |  |  |
| Joyce 2002 | Spatial Working Memory | NART |  |  |  |
| Karch 2009 |  |  |  |  | RT 0-back |
| Katz 2007 | Cognistat |  |  |  |  |
| Keefe 2004 | Logical Memory WMS (STM) | WAIS III information | Letter number sequencing-total | WCST (categories) |  |
| Kerns 2007 | N-Back d’  Sternberg controlled retrieval |  |  |  | Letter search RT |
| Kessler 2007 |  |  |  | NAT |  |
| Kiang 2007 | CVLT II (immediate and Long-Delay free recall) |  |  | WCST (categories) |  |
| Kiefer 2002 | Working Memory Tasks (digit span backward); |  | Category Fluency |  |  |
| Kiehl 2005 |  | NART premorbid; Quick Test |  |  |  |
| Kim 2003 | Delay Recall and immediate recall (Rey-Osterrieth Complex Figure) |  | COWAT (category) | WCST | TMT-A |
| Kim 2004 |  |  |  |  | RT visuospatial maintenance |
| Kircker 2001 |  | NART |  |  |  |
| Kopelowicz 2005 | Verbal Working memory (ACT); immediate memory |  | FAS | WCST (categories) |  |
| Kosmidis 2005 |  |  | Semantic and phonemic task (Total word) |  |  |
| Krabbendam 2000 |  | GIT |  |  |  |
| Kravariti 2003 | General Memory; delayed | IQ |  |  |  |
| Kuperberg 1998 |  | NART premorbid | Letter fluency |  |  |
| Kurachi 1994 | digit span | Picture Completion Test of WAlS |  |  | RT |
| Landro 1993 | Short-Term Memory Test |  |  |  |  |
| Langdon 2002 |  | STW Verbal intelligence |  |  |  |
| Lanser 2002 | Visuo-perceptual Memory | Verbal intelligence |  | Semantic Clustering (CVLT) |  |
| Laplante 1992 |  |  |  |  | Reaction Time |
| Lee 2007 |  |  |  | WCST (categories) |  |
| Leeson 2005 |  | NART premorbid | Picture naming task |  |  |
| Leeson 2009 | RAVLT immediate and long-delay recall | WAIS R o WAIS III |  | CANTAB planning (perfect solutions) |  |
| Leitman 2006 |  | Verbal IQ |  |  |  |
| Leonard 2008 | WJCogR Digit span reversed; STM and LTM | WJCogR Broad Cognitive | WJCogR Verbal analogies |  |  |
| Luck 2008 |  | WAIS-R |  |  | RT |
| MacDonald 2003 | d’context immediate and delayed | WRAT III (raw score) |  |  | AX-CPT |
| Majorek 2009 |  | MWT premorbid |  |  |  |
| Manning 2009 | VPA Verbal memory immediate and delayed | NArt premorbid; FS IQ | COWAT semantic fluency | Stockings of Cambridge |  |
| Martin 2008 |  | WAIS |  |  |  |
| Mathews 2004 |  |  |  |  | Recognition RT |
| Matsui 2008 | Digit span |  | JVLT |  |  |
| McNeely 2003 |  | WAIS-R information |  |  | RT of word identification trials |
| Menzies 2007 | Rivermead Behavioral Memory Test (RBMT) | NART premorbid | Verbal Fluency |  |  |
| Michel 1998 | WMS global index and Delayed recall | WAIS-R |  | WCST n° classements |  |
| Midorikawa 2008 | WMS-R general; verbal memory and delayed | WAIS-R |  | WCST (categories) |  |
| Miller 1995 |  |  | Hard anagrams |  |  |
| Minzenberg 2003 | CVLT Trial 1; Digit span | WAIS-R | Category fluency | MCST |  |
| Mirsky 1995 |  |  | Vocabulary | WCST(categories) | CPT (Reaction Time) |
| Morice 1990 |  | Full IQ | Vocabulary (WAIS-R) | Similarities (WAIS-R) |  |
| Moritz 2001 |  |  |  |  | Prime presentation time with mask (250 ms) |
| Moritz 2005 |  | NART |  |  |  |
| Moritz 2008 |  | premorbid IQ assessed with a vocabulary test |  | targets |  |
| Morrison-Stewart 1991 |  | Wonderlic Personnell Test |  |  |  |
| Mulholland 2008 | RBMT | WAIS-R |  |  |  |
| Muller 2004 | Prose recall immediate  and delayed |  | Word Fluency |  | TMT A |
| Myles-Worsley 1991 |  |  | N’ words |  |  |
| Nestor 1998 | Cued associative recall |  |  |  |  |
| Nestor 2008 | WMS III general; immediate and delayed | WAIS III |  | WCST (categories) |  |
| Neufeld 1978 |  | WAIS-Clarke IQ |  |  |  |
| Neufeld 1995 | Number Named (Spatial Memory) |  |  |  |  |
| Niekawa 2007 | Cognistat Memory |  |  |  |  |
| O’Carroll 1999 |  | Quik Test verbal IQ |  |  |  |
| Ober 1995 |  |  |  |  | Lexical decision task (Reaction Time) |
| Ohrmann 2008 | AVLT immediate and delayed recall | WAIS R vocabulary |  | WCST dynamic correct cards |  |
| Ojeda 2002 |  |  | FAS | WCST (categories) | CPT (reaction time) |
| Okada 2002 |  | WAIS-R |  |  |  |
| Palmer 2010 | Working Memory index |  |  |  |  |
| Papageorgiou 2003 | Digit Span |  |  |  |  |
| Parellada 1994 |  |  |  | WCST (categories) |  |
| Park S 1995 | Memory task (Reaction Time) |  |  |  | RT |
| Park IH 2008 |  | Korean WAIS |  |  |  |
| Paulsen 1994 |  |  | Verbal ability task | Abstraction and cognitive flexibility task |  |
| Penn 1993 |  | Full IQ |  |  | Reaction Time |
| Perlstein 1998 |  |  |  | Card Stroop (color-word association) | Single trial Stroop (reaction time) |
| Perlstein 2001 | Working Memory (Reaction Time) |  |  |  | Target-detection sensitivity |
| Perry 2001 study 1 |  | Vocabolary performance (WAIS-R) |  |  |  |
| Perry 2001 study 3 |  | Vocabolary performance (WAIS-R) |  | WCST (categories) |  |
| Perry 2001 study 4 |  |  |  | WCST (categories) | NAT |
| Phillips 2000 |  | NART |  |  |  |
| Pino 2008 | Verbal Learning test immediate and delayed |  | Verbal fluency |  |  |
| Premkumar 2008 |  | WASI |  |  |  |
| Rabinowicz 1996 |  |  |  |  | RT |
| Rief 1991 |  |  |  | Visual task |  |
| Riley 2000 | Long delay free recall and immediate |  | Verbal Fluency (letters) | WCST (categories) | Trials A psychomotoric speed |
| Rodriguez-Sanchez 2007 | Digit span backward; Verbal Memory | WAIS vocabulary |  |  |  |
| Roesch-Ely 2009 | WM test auditory | MWT premorbid |  |  | RT dual |
| Roiser 2009 | Digit span | WTAR premorbid |  |  | RT high probability |
| Ross 2000 | Accuracy of the cue-location in visuo-spatial and other memory systems | WAIS-R |  |  |  |
| Rossell 1999 |  | NART premorbid | Semantic fluency |  |  |
| Rossell 2008 |  | NART premorbid | Word comprehension task |  |  |
| Rund 2004 |  | WAIS (similarities; picture completion; digit span) |  |  |  |
| Saoud 2000 |  |  |  | WCST (categories) |  |
| Sarfati 1999 |  |  | Character Intention Task (Verbal condition) | Character Intention Task (Image condition) |  |
| Sayers 1995 |  | IQ |  |  |  |
| Schmand 1992 | Verbal learning test (delayed recall) | DART |  |  |  |
| Schreiber 1995 |  | WAIS |  |  | Reaction Time |
| Schuepbach 2002 | Digit Span; WMS-R immediate and delayed |  |  |  |  |
| Schuepbach 2004 | CVLT short delay and long delay recall |  |  |  |  |
| Schwartz 1991 |  |  |  | WCST (categories) |  |
| Seidman 2003 | WMS immediate and delayed recall | WAIS (vocabulary; block design) |  |  |  |
| Sereno 1996 |  |  |  |  | Reaction time in spatial selective attention tasks |
| Shelley 1996 |  |  |  | CPT (hit rate) |  |
| Silver 2003 | Benton Visual Retention Test  digit span | Digit Span (WAIS-R) |  | Executive Function |  |
| Skelley 2008 | WMS Long and short delay | WAIS-R |  |  |  |
| Smid 2009 |  |  |  |  | RT |
| Smith 1998 |  | NART premorbid |  | Stroop test | Trails (B-A) |
| Snyder 2008 | GMLT chase task - STM | NART |  |  |  |
| Soriano 2009 | RIF Rp+ |  |  |  |  |
| Stirling 2001 |  | Quick Test; NART |  |  | RT (CPT) |
| Stirling 2006 | Digit Span (forward+backward) | NART premorbid; Raven Standard Progressive Matrices | FAS | Information Processing speed | RT Stroop Effect |
| Stone 1998 | Non strategic Memory (Recognition); digit span backward |  | Listening Span |  |  |
| Stratta 1999 | Visual-Manual Delayed Response Task |  |  |  | Response time |
| Stratta 2001 |  |  |  | WCST (categories) |  |
| Strik 1993 |  |  |  |  | Oddball paradigm |
| Sullivan 1994 |  | NART |  |  |  |
| Sullivan 2001 |  | NART premorbid; WAIS vocabulary |  |  |  |
| Surguladze 2002 |  | NART premorbid |  |  | RT ipsimodal unrelated |
| Symond 2005 |  |  |  |  | RT stimuli |
| Szoke 2009 |  |  | Verbal Fluency |  |  |
| Tek 2002 | Working Memory Performance |  |  |  | Target exposure durations |
| Tendolkar 2002 | AVLT (delayed and immediate recall) |  | Verbal Fluency |  | CPT |
| Thomas 1996 | Reverse Digit Span Test (Working Memory) |  |  |  |  |
| Torres 2007 |  |  |  | EPL |  |
| Tsoi 2008 |  | NART |  |  |  |
| Ueno 2004 |  |  |  |  | RT |
| Van Beilen 2004 | 15-Word Test |  | Fluency test (derived from GIT) |  |  |
| Van Den Bosch 1992 | span 10 letters |  |  |  | CPT (percentage correct responses and reaction time) |
| Van Erp 2008 | Recognition accuracy |  |  |  |  |
| Verdoux 1994 | Rey complex figure test (Rey recall) |  | Verbal fluency test | WCST (categories) |  |
| Vinogradov 2002 |  | WAIS-R | Lexical decision |  | RT |
| Wang 2008 | WMS delay and immediate | WAIS-R | Verbal fluency | WCST (categories) |  |
| Waters 2006 | Digit span backward (WAIS III) | NART premorbid |  |  |  |
| Weickert 2000 | WMS Logical memory | WAIS | Verbal fluency test | WCST (categories) |  |
| Weiler 2009 | WMS Digit Span |  |  |  |  |
| Weiss A.P. 2002 | Old/new recognition memory test |  |  |  |  |
| Weiss K.M. 1992 study 1 |  |  |  |  | Two-flash fusion (critical duration) |
| Wexler 1998 | 16 words immediate recall |  |  |  |  |
| Wilk 2002 | RBANS (Delayed and immediate Memory) |  | RBANS |  |  |
| Wood 2006 | Digit span; AMIPB Delayed and immediate recall | NART Premorbid QI |  |  |  |
| Woonings 2002 | Secondary verbal memory (RAVLT recall) |  |  | WCST (categories) |  |
| Yogev 2004 |  |  |  | CAT N° of categories completed |  |
| Zuffante 2001 |  | IQ (performance) |  |  | Spatial Delay response Task |

Legend:

ACT (Auditory Consonant Trigram);AMIPB (Adult Memory and Information Processing Battery); AMIPB (Adult Memory and Information Processing Battery); AVLT (Auditory Verbal Learning Test); CAL (Conditional Associative Learning); CANTAB (Cambridge Neuropsychological Test Automated Battery); CAT (Common Admission Test); CERAD (Consortium to Establish a Registry for Alzheimer’s Disease); COGNISTAT (Neurobehavioral Cognitive Status Examination-NCSE); COWAT (Controlled oral word association test); CPT (Continuous Performance Test); CVLT (California Verbal Learning Test); DART (Dutch Adult Reading Test); DMTS (Delayed Match Sample); EPL (Piaget-Longeot Logical Thought Evaluation Scale); FAS (Phonological Fluency); GIT (Groningen Intelligence Test); GMLT (Groton Maze Learning Test); JVLT (JapaneseVerbal Learning Test); MCST (Modified Wisconsin Card Sorting Test); MVPT-V (Motor-Free Visual Perception Test - Vertical Format); MWT (Mehrfachwahlwortschatztest); NART (National Adult Reading Test); NAT (Numerical Attention Test); RAVLT (Rey Auditory Verbal Learning Test); RBANS (Repeatable Battery for the Assessment of Neuropsychological Status); RBMT (Rivermead Behavioural Memory Test); RIF (Retrieval Induced Forgetting); RT (reaction time); SILS (Shipley Institute of Living Scale); SOC (Stocking of Cambridge); SRM (Spatial Recognition Memory); STW (Spot the word); SVLT (Semantic Verbal Learning Test); TBR (“to be remembered” item); TMT (Trail Making Test); VPA (Verbal Paired Associates); WAIS (Wechsler Adult Intelligence Scale); WASI (Wechsler Abbreviated Scale of Intelligence); WCST (Wisconsin Card Sorting Test); WJCogR (Woodcock–Johnson Test of Cognitive Ability); WMS (Wechsler Memory Scale); WTAR (Wechsler Test of Adult Reading).
